# Plasmonic Pt nanoparticles—TiO_2_ hierarchical nano-architecture as a visible light photocatalyst for water splitting

**DOI:** 10.1038/s41598-018-33795-z

**Published:** 2018-11-01

**Authors:** Lipei Qin, Guojing Wang, Yiwei Tan

**Affiliations:** State Key Laboratory of Materials-Oriented Chemical Engineering, School of Chemistry and Chemical Engineering, Nanjing Tech University, Nanjing, 210009 China

## Abstract

Visible light-driven water splitting (VLWS) into hydrogen and oxygen is attractive and depends on efficient photocatalysts. Herein, we demonstrate the first exploration of the capability to control the morphology of nanostructured TiO_2_ in conjunction with the choice of a suitable plasmonic metal (PM) to fabricate novel photocatalysts that are capable of harvesting visible light for more efficient VL-fuel conversion. This methodology affords us to successful access to the novel plasmonic Pt/TiO_2_-HA (large Pt nanoparticles (NPs) supported on TiO_2_ hierarchical nano-architecture (TiO_2_-HA)) photocatalysts that exhibit plasmon absorption in the visible range and consequent outstanding activity and durability for VLWS. Particularly, the Pt/TiO_2_-HA shows an excellent photocatalytic activity for overall water splitting rather than only for hydrogen evolution (HE), which is superior to those of the conventional plasmonic Au/TiO_2_ photocatalysts. The synergistic effects of the high Schottky barrier at the Pt–TiO_2_-HA interface, which induces the stronger reduction ability of hot electrons, and intrinsic Pt catalytic activity are responsible for the exceptional photocatalytic performance of Pt/TiO_2_-HA and simplify the composition of plasmonic photocatalysts.

## Introduction

Design and fabrication of high-performance photocatalysts with suitable architectures for VLWS into hydrogen and oxygen has been a topic of tremendous scientific interest in recent years because this process is promising for resolving today’s increased global environmental crisis and energy shortage^1–4^. The exploration of highly efficient photocatalysts with a strong response to visible light, a high activity and stability, and low cost is critical to construct an artificial leaf for realizing solar energy conversion. Among various promising photocatalysts for water splitting, TiO_2_ is an ideal candidate and has received much more attention than all other known efficient ones owing to its unique and diverse favorable properties^5^, such as a facile manipulation of its nanoscale morphology, excellent long-term chemical stability, robust anti-photo-corrosion ability endowed by strong chemical bonding between Ti(IV) and O ions, low toxicity, and easy availability due to its Earth-abundance. However, the conversion efficiency of TiO_2_ still remains at a very low level because of its limited absorption of solar radiation (*λ* < 400 nm) and the intractable recombination of photogenerated charge carriers in it.

Notably, a variety of methods, including doping with various elements^6–8^, engineering nano-heterostructures with semiconductor quantum dots or metal oxides^9–12^, and combining with PM-NPs (typically, Au and Ag)^13–31^, have been reported to effectively enhance visible light harvesting of TiO_2_ and concomitantly promote carrier separation and transfer. In particular, many PM-NPs/TiO_2_ composites have shown remarkably plasmon-enhanced photocatalytic activities towards VLWS by extending light absorption to visible region, which is induced by greatly enhancing local electric field at the interface of PM-NPs and TiO_2_ or by injecting hot electrons from photo-excited PM-NPs to TiO_2_ via surface plasmon resonance (SPR) effects of the supported PM-NPs^13–30^. Therefore, advanced strategies for producing nano-architectures consisting of PM-NPs and TiO_2_ have been actively pursued by world-wide researchers because of the extraordinarily high stability of Au and Ag and the fascinating underlying physical mechanisms.

However, Au and Ag NPs show very low, and even not, chemical catalytic activities for water splitting. Concomitantly, the coverage of these PM-NPs could significantly diminish the catalytically active surface area^28^. These drawbacks generally leads to the Au or Ag NPs-decorated semiconductor photocatalysts showing activity towards a half reaction of water splitting producing only H_2_ or O_2_ with the need of sacrificial regents (SR)^18–25^ or additional driving bias^26–28,32,33^. To date, there have been very few reports on photocatalysts used for the full VLWS (i.e., SR-free and unbiased)^29,30^. Nonetheless, the activities of those plasmonic Au NPs-based multi-component photocatalysts are still too low to meet the challenging demands of renewable fuel production^29,30^. In addition, the synthesis of TiO_2_ nanostructures still remains challenging to improve light absorption and suppress the recombination events. In contrast, platinum shows the highest catalytic activity for hydrogen evolution (HE) and moderate activity for oxygen evolution (OE). So far, only small Pt NPs that merely serve as a chemical catalyst are loaded on TiO_2_ to facilitate HE^21,22,29,30,34^. The plasmonic absorption of Pt NPs typically in the UV region has been the main obstacle for use of Pt NPs as a plasmonic enhancement agent.

Most recently, Bigall *et al*. have established some very effective methods to extend the localized surface plasmonic (LSP) absorption of Pt NPs to the visible and even near-IR regions of spectrum by enlarging their size^35–37^. The reported Pt NPs exhibit strong and broad plasmonic absorption features. In the report of Bigall *et al*., the plasmonic absorption maximum can be tuned from ~400 to 494 nm by increasing Pt NP size from 73 to 107 nm^35^. Jung and co-workers prepared uniform Pt nanorods showing a transverse LSP mode appearing at ~400 nm and a longitudinal mode redshifting from 800 to above 1600 nm with increasing the length^36^. In addition, the LSP features of dimeric Pt NPs are different from a single NP due to plasmonic coupling resulting in dramatic redshift (from 650 to 750 nm), enhancement, and broadening of LSP peak^37^. Inspired by these investigations, we aim to study the ability and functionality of large plasmonic Pt NPs in photosensitization of semiconductor. As a result, to overcome the drawbacks of PM-NPs/TiO_2_, herein, we develop a facile strategy to obtain a new plasmonic Pt/TiO_2_-HA heterostructure by fabricating the novel TiO_2_-HA (TiO_2_ branched nanowires (b-NWs) epitaxially grown on TiO_2_ microtubes) decorated by large Pt NPs with SPR in the visible region, which enables us to fulfill a dual function, i.e., control over photosensitivity and improving surface catalytic function with the aid of the plasmonic Pt NPs. Thus, photocatalytic overall water splitting can be realized without using any SR and additional co-catalysts. In particular, it should be stressed that to realize the practical application of VLWS, the development of photocatalysts working in a system free from any chemicals except the photocatalyst and water, as in this work, is much more desirable than the exploration of conventional photocatalysts in combination with sacrificial agents, hole consumers, or scavengers^38–41^.

## Results and Discussion

### Morphological and structural characterizations of TiO_2_-HA and Pt/TiO_2_-HA

**Figure 1:**
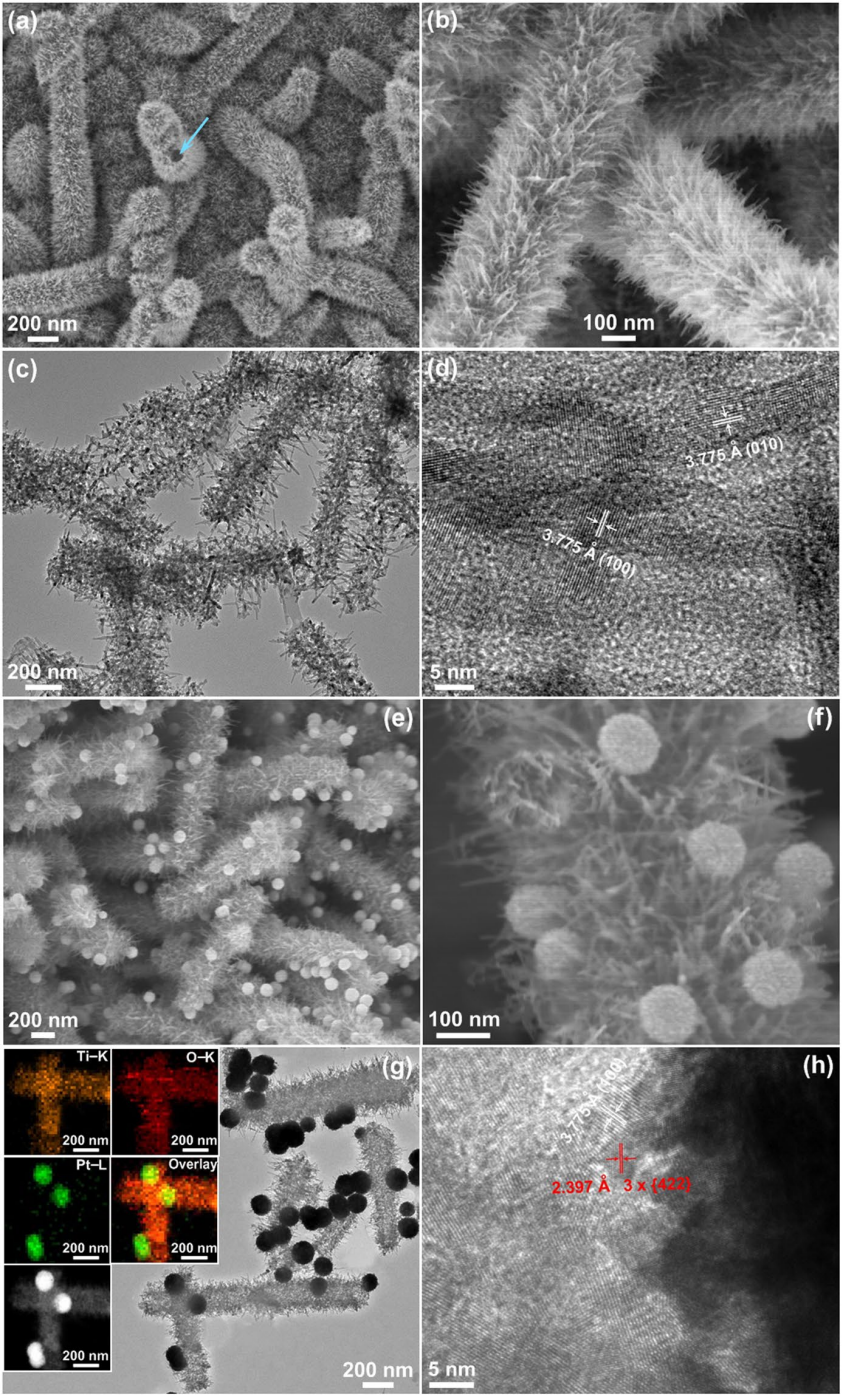
(**a** and **e**) Low- and (**b** and **f**) high-magnification SEM, (c and g) TEM, and (d and h) HRTEM images of (**a**–**d**) the TiO_2_-HA and (**e**–**h**) the Pt107-7/TiO_2_-HA with 7 wt% loading of Pt NPs. The arrow in panel (a) points to the cavity of a broken hollow TiO_2_-HA. The insets: (**b**) SEM image showing the cross sections of several TiO_2_-HAs; (**g**) EDS elemental mapping images of Ti, O, and Pt, their overlay image, and the corresponding HAADF-STEM image (the bottom inset in panel (g)).

The TiO_2_-HA is produced by hydrolysis of K_2_TiO(C_2_O_4_)_2_ in the presence of the additive NaH_2_PO_2_ and capping regent diethylene glycol at 180 °C under solvothermal conditions. NaH_2_PO_2_ serves as a structure-directing agent to control the morphology of TiO_2_-HA (see Figs S1 and S2, and the related discussion in the Supporting Information (SI)). The overview scanning electron microscopy (SEM) image in Fig. 1a unambiguously displays that the TiO_2_-HA is produced in a 100% morphological yield. The characteristic feature of the TiO_2_-HA is that very thin, highly dense TiO_2_ b-NWs are radially (epitaxially) grown from the whole length of the primary TiO_2_ microtube trunk with a length of 0.5‒2 μm (see Fig. 1b,c and the discussion below). The transmission electron microscopy (TEM) image in Fig. 1c presents that the b-NWs have a length and a diameter in the range of 100‒200 and 4‒10 nm, respectively. In particular, the TEM image in Fig. 1c reveals that each TiO_2_ trunk has a hollow interior with a shell thickness of 20‒30 nm, as evidenced by the different contrasts between the middle and the two sides (also see Fig. 1g). The broken TiO_2_-HA shown in Fig. 1a also corroborates the tubular superstructure of each trunk. All the TiO_2_-HAs have a complete microtube trunk with both closed ends of which one is round and the other is flat (Fig. 1a and the inset in Fig. 1b). The outer diameter of the microtube trunks ranges from 150 to 250 nm. The ultrathin TiO_2_ b-NWs provide a high specific surface area (S_BET_ of 150.2 m^2^ g^‒1^, Fig. S3) for harvesting light, short distances for migration of charge carries, and a large contact area with electrolyte, while the large central trunks enhance light scattering. These features of TiO_2_-HA result in a significant extension of the light travelling distance within the ensemble of the TiO_2_-HAs and therefore increase the probability of photons being absorbed by TiO_2_-HA. Such a binary synergistic function of the different parts of TiO_2_-HA could greatly promote its photocatalytic capability.

**Figure 2:**
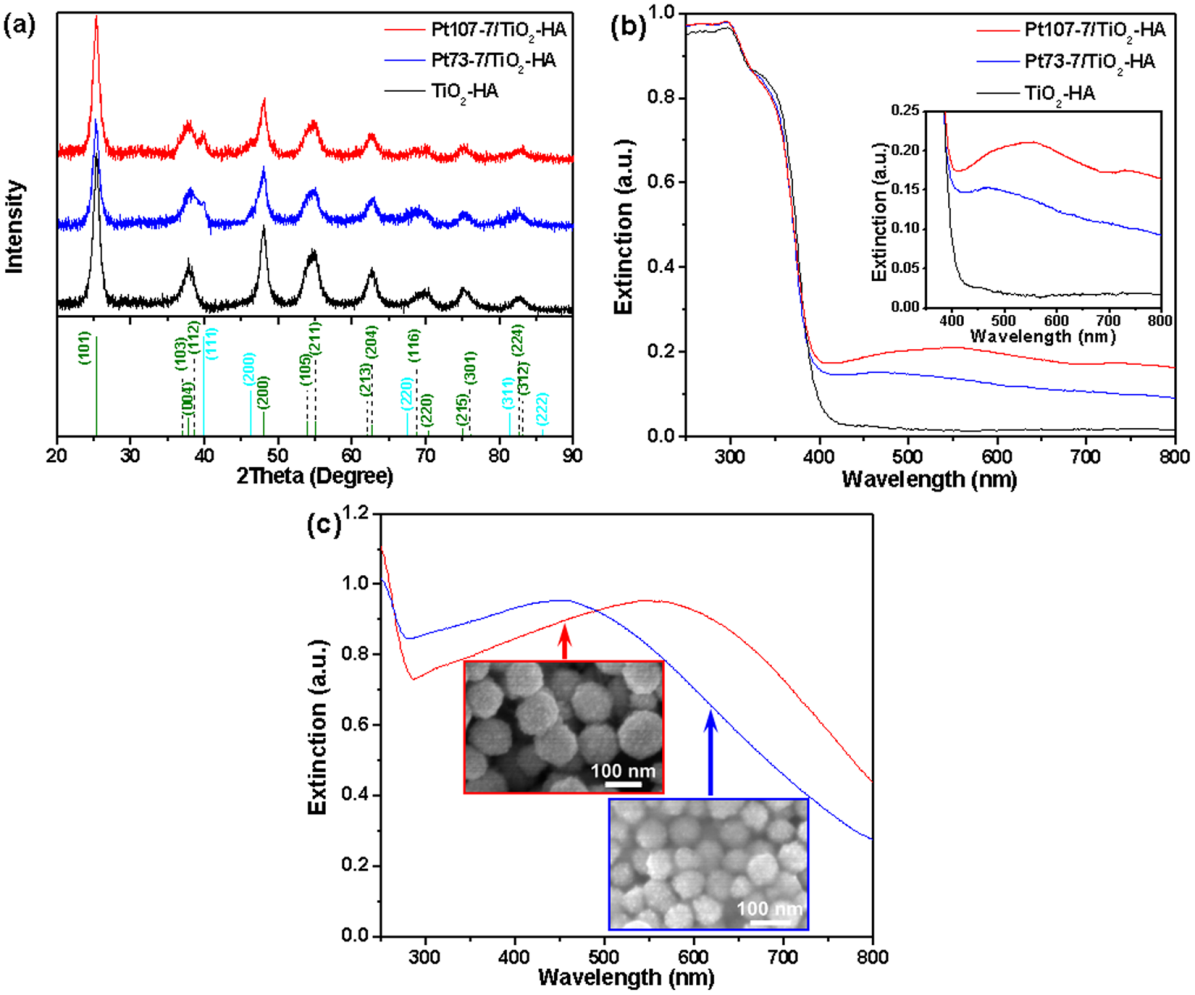
(**a**) XRD patterns of the TiO_2_-HA, Pt73-7/TiO_2_-HA, and Pt107-7/TiO_2_-HA. For comparison, the intensities and positions for the pure TiO_2_ (olive bars, JCPDF No. 78-2486) and Pt (cyan bars, JCPDF No. 87-0646) references are given at the bottom according to the JCPDS database. UV-vis extinction spectra of (**b**) the TiO_2_-HA, Pt73-7/TiO_2_-HA, and Pt107-7/TiO_2_-HA and of (**c**) the Pt NPs with a mean diameter of 73 (blue curve, *λ*_max_ = 455 nm) and 107 nm (red curve, *λ*_max_ = 542 nm). The insets: (**b**) a zoom-in view of optical extinction in the visible region; (**c**) SEM images of the corresponding Pt NPs.

The high resolution TEM (HRTEM) image in Fig. 1d shows the well-resolved, continuous lattice fringes with the same spacing, indicating that each branch is single crystalline and has the same growth direction. The *d*-spacing of adjacent fringes along the axial direction of the b-NWs is consistently 3.775 Å, corresponding to the (100) planes of anatase phase, suggesting that all the b-NWs of the TiO_2_-HA epitaxially grow exclusively along [100] directions. The XRD pattern in Fig. 2a illustrates that all the Bragg peaks of the TiO_2_-HA can be perfectly indexed to pure anatase phase of TiO_2_ with lattice constants *a* = 3.784 Å and *c* = 9.514 Å (space group: I41/amd (141), JCPDF no. 78-2486). Furthermore, the strong diffraction peaks confirm the good crystallinity of the TiO_2_-HA. The surface chemical composition and electronic state of the TiO_2_-HA are further analyzed using X-ray photoelectron spectroscopy (XPS). The XPS data demonstrate the expected elements Ti(IV) and O species for TiO_2_ (Fig. S1 and the related discussion in SI).

To improve visible light harvesting of the TiO_2_-HA, the uniform Pt NPs with a mean diameter of 73 or 107 nm were directly grown on the TiO_2_-HA. Correspondingly, the integrated composites of the TiO_2_-HA and 73 or 107 nm Pt NPs are designated as Pt73/TiO_2_-HA or Pt107/TiO_2_-HA, respectively. The Pt NPs loading is varied from 1% and 3% to 7% by weight with respect to TiO_2_-HA. For simplicity, the composite samples will be referred to Pt73-x/TiO_2_-HA and Pt107-x/TiO_2_-HA in the following, where x represents the weight percentage of Pt NPs, e.g., Pt107-7/TiO_2_-HA refers to the Pt107/TiO_2_-HA containing 7 wt% 107 nm Pt NPs. The structural characterization results for Pt107/TiO_2_-HA and Pt73/TiO_2_-HA are very similar and therefore for brevity we mainly focus on the Pt107/TiO_2_-HA and the results of the Pt73/TiO_2_-HA are not shown. SEM images show the homogeneous distribution of the ~107 nm Pt NPs over the TiO_2_-HA and verify that the number density of Pt NPs on TiO_2_-HA increases with Pt NPs loading (Figs 1e,f and S4a,b). Representatively, the TEM image of the Pt107-7/TiO_2_-HA in Fig. 1g reveals that Pt NPs are slightly embedded in the spacings among a number of b-NWs, as evidenced by the relatively slight contrast in the contact region between Pt NPs and the TiO_2_-HA compared to other domains of NPs. Such a embedding configuration could be advantageous for electric field enhancement and more efficient transfer of hot electrons^19,24,42^. The corresponding energy dispersive X-ray spectroscopy (EDS) elemental-mapping data in the insets in Fig. 1g and EDS spectrum in Fig. S4c support that the TiO_2_-HA is comprised of Ti and O atoms and decorated with Pt NPs. The quantitative EDS analysis also confirms the concentration of Pt NPs in each composite sample is consistent with the initial designated level (Table S1). In addition, the enhanced elemental contrast shown by the high annular dark-field scanning TEM (HAADF-STEM) image also implies that Pt NPs were successfully loaded on TiO_2_-HA (the bottom left inset in Fig. 1g).

Representatively, the close-up HRTEM image in Fig. 1h shows the interfacial area of the Pt107-7/TiO_2_-HA composite. Two sets of distinctive lattice planes, which can be assigned to anatase and Pt, can be easily discerned. The well-resolved (100) planes of the continuous TiO_2_ shell domain can be clearly identified, which proves the epitaxial growth of all the b-NWs along [100] directions. The lattice fringes observed in the margin of Pt NPs correspond to a periodicity of 2.40 Å, which can be ascribed to (1/3){422} forbidden reflections. Such forbidden reflections have been frequently observed for fcc Au and Ag NPs that are grown by seeded methods and can be attributed to the generation of twins and stacking faults in the fcc lattice^43^. For comparison, the XRD patterns of the Pt107-7/TiO_2_-HA and Pt73-7/TiO_2_-HA present the same reflection peaks originating from TiO_2_ and Pt constituents (Fig. 2a), further confirming the formation of the Pt/TiO_2_-HA composite. However, no definitive diffraction peaks from Pt NPs can be identified for the Pt107/TiO_2_-HA and Pt73/TiO_2_-HA samples when the content of Pt is lower than 5 wt%. The formation of metallic Pt in Pt107-7/TiO_2_-HA composite is further verified by XPS analysis (Fig. S5).

Figure 2b compares the extinction spectra of the TiO_2_-HA, Pt73-7/TiO_2_-HA, and Pt107-7/TiO_2_-HA. As expected, the pure TiO_2_-HA sample exhibits bandgap-dependent absorption only in the UV region (*λ* < 400 nm). The extended tail beyond 400 nm in the TiO_2_-HA extinction spectrum stems from its light scattering behavior rather than intrinsic optical absorption (see discussion in the later section). In contrast, the Pt107-7/TiO_2_-HA sample exhibits a pronounced, broad optical absorption hump in the visible region. For comparison, large Pt colloids with a similar diameter of 73 or 107 nm, which were prepared according to the procedure reported by Bigall *et al*.^35^, show a plasmonic absorption peak at 455 or 542 nm, respectively (Fig. 2c). It is worth noting that compared to the plasmonic absorption of the corresponding colloidal Pt NPs, the extinction bands of the Pt73-7/TiO_2_-HA and Pt107-7/TiO_2_-HA are red-shifted by 11 and 13 nm, respectively. Furthermore, the band of the Pt107-7/TiO_2_-HA is broadened and extended even further to the near-infrared (NIR) region (*λ* > 700 nm, the inset in Fig. 2c). These changes in the extinction spectra, especially for the Pt107-7/TiO_2_, can be attributed to a superposition of the enhancement of local electric field near the interface between the Pt NPs and TiO_2_-HA and scattering of photons due to the larger Pt NPs^19,24^. Obviously, similar to the Au NPs^19,22^, the bigger Pt NPs, the larger near field enhancement at the Pt NP‒TiO_2_-HA interface because the SPR intensity increases with particulate size. For both the Pt NPs-supported samples, their absorption intensity increases in the visible region with the concentration of supported Pt NPs in our experimental range (Fig. S6).

### Photocatalytic performance of Pt/TiO_2_-HA

**Figure 3:**
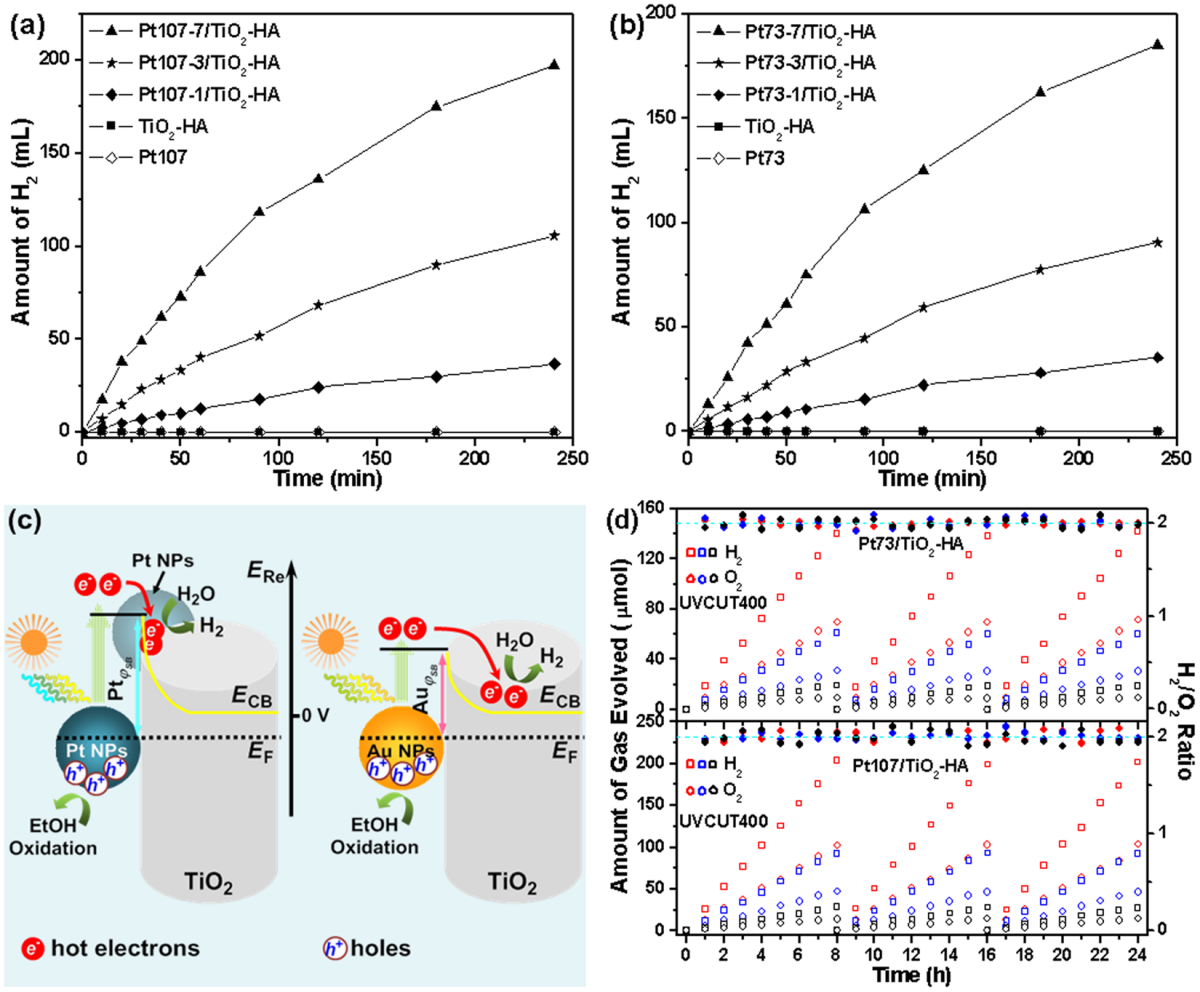
Time course of evolved H_2_ from a 25 vol% water/ethanol system under visible light (*λ* > 400 nm) irradiation in the presence of (**a**) the Pt107/TiO_2_-HA and (**b**) the Pt73/TiO_2_-HA photocatalysts containing varied Pt NPs loading. (**c**) Schematic illustration of *φ*_SB_-dependent hot electron generation and transfer from Pt or Au NPs to TiO_2_. *E*_F_, *E*_CB_, and *E*_Re_ represent the energies of the Fermi level, TiO_2_ conduction band, and chemical reduction potential, respectively. For clarity, the exciting lights are labeled as different colors approximately corresponding to the varied colored light absorbed by the plasmonic metal NPs. (**d**) Photocatalytic H_2_ and O_2_ evolution from pure water in 3 repeated cycles (8 h/cycle) using various Pt/TiO_2_-HA (data point: red, Pt107-7 and Pt73-7; blue, Pt107-3 and Pt73-3; black, Pt107-1 and Pt73-1) photocatalysts under visible light (*λ* > 400 nm) irradiation. The testing system was evacuated between two cycles in turn.

Figure 3a,b illustrate the rates of photocatalytic HE from an aqueous solution of ethanol (4: 1 water/ethanol in v/v) for various Pt/TiO_2_-HAs and pure TiO_2_-HA as a function of time under visible light (*λ* > 400 nm) illumination. The utilization of a conventional sacrificial agent (ethanol) for evaluating the photocatalytic activity of various Pt/TiO_2_-HAs provides a consistent comparison with those results from the Au/TiO_2_ photocatalysts in the literature. Pt NPs and TiO_2_-HA do not exhibit any activity (Fig. 3a,b), indicating that TiO_2_-HA exhibits no optical absorption in the region of *λ* > 400 nm. No HE is also detected over the TiO_2_-HA decorated by small Pt NPs with ~25 nm in size (Fig. S7), which enables adequate suppression of the e−h pair recombination in TiO_2_-HA, further confirming that TiO_2_-HA exhibits no response to visible light. All of the Pt107/TiO_2_-HA samples present a higher HE rate and a larger mass activity (MA) than the corresponding Pt73/TiO_2_-HA sample with the same Pt NP loading (also see Table S2). Therefore, the 107 nm Pt NPs with a larger field enhancement appears to further promote the photocatalytic activity. For the samples with Pt loading not less than 3 wt%, their total MAs are much higher than those of the Au/TiO_2_ photocatalysts reported previously (Table S2). Notably, the PM-based MAs of all the samples are at least 3-fold higher than the previous Au/TiO_2_ (Table S2). The greater photocatalytic activity achieved by the Pt/TiO_2_-HA indicates that the plasmonic Pt NPs could play an important role in enhancing photocatalysis besides the favorable TiO_2_-HA morphology discussed above. In particular, unlike the Au/TiO_2_ photocatalysts (a volcano curve for activity versus Au loading)^28^, the HE rate monotonously rises with Pt loading within our experimental range, suggesting that the activity sites simultaneously increase because Pt also acts as a highly active HE catalyst. The PM-based MA of each Pt/TiO_2_-HA with the same Pt size approves this proposition because it does not differ much by altering Pt loading (Table S2).

**Figure 4:**
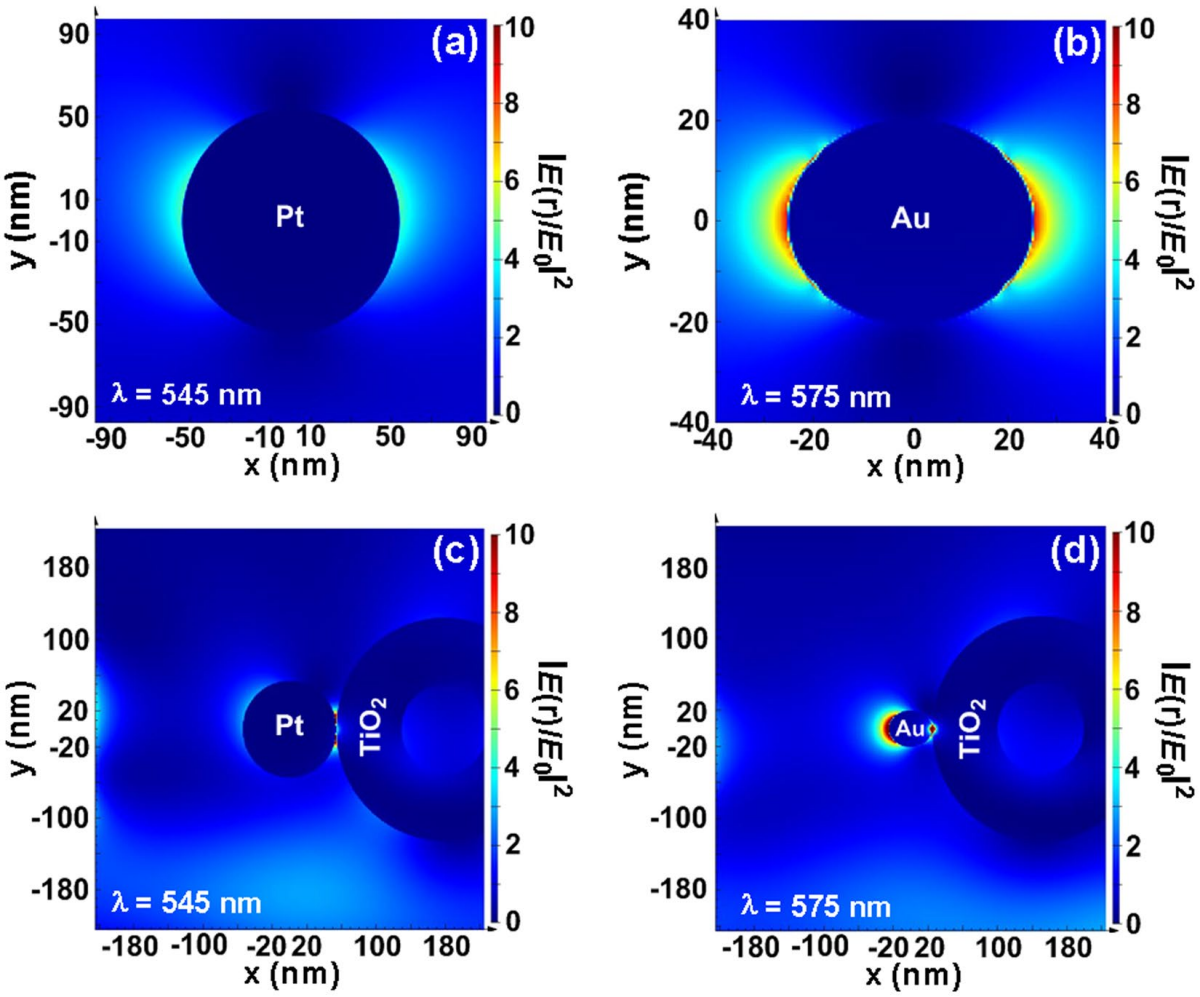
Plasmonic near-field distribution maps (cross-section view at *z* = 0) of (**a**) Pt107 nanospheres and (**b**) ellipsoidal Au NPs with major axis of 60 nm and minor axis of 40 nm simulated using finite difference time domain methods, which show the evanescent electric near-field intensity enhancements |*E*(r)/*E*_0_|^2^ of the Pt and Au NPs at 545 and 575 nm, respectively. Electric field intensity enhancement contours of (**c**) the Pt107/TiO_2_-HA and (**d**) Au_50 nm_/TiO_2_-HA hybrid systems under the parallel polarized excitation. The color scale bar on the right side of each panel shows the relative increase in field enhancement. The geometrical shapes of the Pt107, Au_50 nm_, and TiO_2_-HA are established based on the TEM images.

In the case of Au/TiO_2_ photocatalysts, their enhanced activities endowed by Au NPs have recently been elucidated by the following mechanisms: (i) the enhanced optical absorption of TiO_2_ driven by SPR-magnified electromagnetic fields, (ii) SPR-induced hot electron injection from Au NPs to TiO_2_, and (iii) resonant photon scattering^22,28^. However, the nature is different in the plasmonic Pt/TiO_2_ photocatalysts. First, the plasmonic field enhancement from Pt SPR is lower than that from Au and Ag counterparts^35^. In our case, obviously, the corresponding optical-absorption maps display that the Pt107 NPs exhibit a much weaker plasmonic near-field than the Au_50 nm_ NPs (i.e., Au NPs with a mean diameter of 50 nm and an ellipsoidal shape to conform to the following synthetic results), as evidenced by performing numerical simulations using the finite-difference time-domain (FDTD) method (Fig. 4a,b). To further compare the nature of the plasmon mode at the Pt107/TiO_2_-HA interface with that at the Au_50 nm_/TiO_2_-HA interface, the FDTD simulations were conducted to calculate the electric field enhancement at each interface, in which parallel incident light polarization direction is applied to the Pt107 and Au_50 nm_ NPs. The electric field intensity enhancement contour reveals that the enhancement of the local electric field is mediocre at the Pt107/TiO_2_-HA interface, whereas a spatially confined “hot spot” appears at the Au_50 nm_/TiO_2_-HA interface (Fig. 4c,d). Therefore, the local electric field enhancement has insignificant impacts on the photocatalytic activity of the Pt107/TiO_2_-HA system. Second, the Schottky barrier height (*φ*_*SB*_) of Pt/TiO_2_ junction (*φ*_*SB*(Pt/TiO2)_ = 1.7 eV)^44,45^ is higher than that of Au/TiO_2_ (*φ*_*SB*(Au/TiO2)_ = 0.9–1.0 eV)^46^. These two unique physical properties of Pt/TiO_2_ suggest that the photocatalytic HE process for the Pt/TiO_2_-HAs is mechanistically different from that of Au/TiO_2_ photocatalysts. Despite the relatively low field enhancement afforded by the supported Pt NPs and no optical absorption for TiO_2_-HA in the visible region, all the Pt/TiO_2_-HA samples show a much higher PM-based MA than the best Au-TiO_2_ photocatalysts reported by different groups^19,24^. Therefore, the mechanism of local enhanced electric field at the Pt–TiO_2_-HA interface should not be responsible for the higher photocatalytic activity of the Pt/TiO_2_-HA. In addition, the increase in absorbed photons scattered by TiO_2_-HA itself and large Pt NPs plays a certain but not dominant role in boosting photocatalytic activity.

On the other hand, the larger *φ*_*SB*(Pt/TiO2)_ results in the hot electrons transferred from the Pt NPs to occupy the higher energy levels in the TiO_2_-HA conduction band (CB) in comparison with Au/TiO_2_ photocatalysts, giving rise to the more negative potential of the accumulated hot electrons (see Fig. 3c). This is because the higher *φ*_*SB*(Pt/TiO2)_ at the Pt–TiO_2_-HA interface effectively prevents the injected hot electrons from striding over the Schottky barrier and transferring back to recombine with the holes left within Pt NPs and thereby allows the hot electrons to survive on a very long time scale. Thus, compared to Au/TiO_2_, the increased population of hot electrons due to their prolonged lifetimes increases the probability of the injected electrons to occupy the higher energy levels in the TiO_2_-HA CB and enhances their reduction potentials (Fig. 3c)^22^. This hypothesis is strongly supported by monitoring the open circuit voltage decay versus time characteristics, as recently introduced by DuChene *et al*.^47^, which reveals the longer hot electron lifetime for the Pt107-7/TiO_2_-HA electrode than that for the Au_50 nm_/TiO_2_-HA (i.e., 50 nm Au NPs-decorated TiO_2_-HA (8.3 wt% Au)) (Fig. S8 and the related discussion in SI). The energy of SPR in Pt NPs is sufficiently high and can be much larger than the *φ*_*SB*(Pt/TiO2)_ (1.7 eV). For example, it is estimated to be 3.26 eV based on the recent report of Lin *et al*.^48^. In our case, the SPR absorption maxima of the Pt73 and Pt107 correspond to the photon energy of 2.7 and 2.3 eV, respectively. At the same time, the plasmonic hot electrons generated by our Pt NPs are excited by a large fraction of shorter wavelength light compared to Au/TiO_2_. Accordingly, our experimental results suggest that the excited carriers are energetic and momentum enough to surmount the Pt–TiO_2_-HA interface, so that a sufficient amount of the hot electrons can be injected into the TiO_2_-HA CB and accumulated there to increase reduction potentials that enable the higher water reduction rate than that on Au/TiO_2_.

Meanwhile, the Pt NPs with intrinsic excellent catalytic activity also significantly raise the rate of water photoreduction. Among various catalysts, Pt exhibits the best catalytic activity towards HE (i.e., the overpotential close to zero for H_2_ formation and facile H_2_ desorption) and thus the most favorable HE kinetics and a very high energetic efficiency. Therefore, the recombination of a considerable portion of the hot electrons in the TiO_2_-HA CB and holes on Pt NPs can be further suppressed by the extremely high water reduction rate (i.e., rapid consumption of electrons at more negative potentials) considering the competition between hot electrons reducing water and recombining with those holes. In Fig. 3c, for clarity, an additional Pt NP is schematically depicted to highlight the HE reaction facilitated by coupling of the catalytic function of the plasmonic Pt NPs and the favourable reduction potentials of the hot electrons. Concomitantly, the injected hot electrons are inclined to transfer from their original host NP into other guest Pt NPs by tunneling due to the electron migration driven by the repulsive forces that are induced by the accumulated electrons (i.e, the electric field gradient) in the TiO_2_-HA CB^21,49^, and moreover, other Pt NPs are more accessible to the injected hot electrons than the original host NP (namely, the lower possibility of hot electrons diffusing back to the host NP) because of their larger population. In contrast, Au shows inappreciable catalytic activity towards HE, so that only a small part of hot electrons are able to participate in the water photoreduction, leading to the lower HE rates. Meanwhile, the hot electrons rapid relax from the high energy states determined by the *φ*_*SB*(Au/TiO2)_ to the level of the conduction band minimum and eventually the majority of hot electrons recombine with the holes on Au NPs^47,50^. Unlike the plasmonic Pt/TiO_2_-HA, water photoreduction proceeds on the surface of the TiO_2_ moiety of Au/TiO_2_ because of the transferred hot electrons lying in the TiO_2_ CB and the catalytically inactive “by-stander” Au moiety.

To further confirm our new mechanism, two control samples including Au_50 nm_/TiO_2_-HA and Au_50 nm_/TiO_2_-HA–Pt_25 nm_ (i.e., Au_50 nm_/TiO_2_-HA containing 25 nm Pt NP co-catalyst (6.8 wt%)) were prepared and used as photocatalysts for water reduction under otherwise the same conditions as those used in testing of the Pt/TiO_2_-HA (Figs S9, S10, and S11a). It should be mentioned that all of the TiO_2_-HA supports show nearly the same absorption spectra, which lays a solid foundation to make a reliable comparison among photocatalytic activities of different samples (Figs 2b and S10b). In the case of the Au_50 nm_/TiO_2_-HA–Pt_25 nm_, the incorporation of small Pt NPs provides the comparable chemical catalytic effect on HE to that of the Pt107-7(Pt73-7)/TiO_2_-HA such that the net hot-electron effect can be exactly extracted from the Pt/TiO_2_-HA. The PM-based MAs of all the Pt/TiO_2_-HA samples are not only much higher than the control samples, especially the Au_50 nm_/TiO_2_-HA–Pt_25 nm_ (Table S2) but also essentially independent of Pt NP loading, which further validates the mechanism of hot electron reduction regulated by the Pt–TiO_2_-HA interface.

Concomitantly, for our plasmonic Pt/TiO_2_-HA samples, the population of holes with more positive potentials is expected to increase in comparison with Au/TiO_2_, presumably due to the higher plasmonic energy obtained from a portion of exciting light with higher photon energies (Fig. 3c). On the other hand, the presence of large Pt NPs with small specific surface areas is favourable for concentrating charge carries (i.e., holes) in the vicinity of NP surface, that is to say, increasing the surface concentration of holes, because an order of magnitude increase in surface area can diminish the obtainable photovoltage by 59 mV^1,51^. It can be deduced that the potential of a fraction of the accumulated holes on the large Pt NPs can be higher than the value of *ca*. 1.6 V (*vs*. reversible hydrogen electrode) required to achieve the onset of water oxidation on Pt^52^. In addition, the sufficiently high *φ*_*SB*(Pt/TiO2)_ can effectively inhibit the recombination of the hot electrons in the TiO_2_-HA CB with the holes left on the Pt NPs and thus permits the holes to accumulate and occupy the higher energy levels in the valence band of Pt based on the time scale of the hot electron lifetime discussed above, which also contributes to the enhanced oxidation ability. Therefore, this increases the open circuit photovoltage within photocatalyst particles by decreasing the photocatalyst/electrolyte interfacial area, leading to the improvement of water oxidation rate. A further conclusion can be deduced from the experimental results discussed below.

The evaluation of photocatalysts for practical applications requires that photocatalytic activity should be measured in pure water without any sacrificial agents. Interestingly, significant gas generation from pure water is observed over the Pt/TiO_2_-HA samples under visible (*λ* > 400 nm) light irradiation. Figure 3d depicts the time courses of HE and OE obtained in a suspension of each Pt/TiO_2_-HA photocatalyst in pure water. There is a proportional increase in the amounts of H_2_ and O_2_ with irradiation time within each illumination cycle and H_2_/O_2_ ratio is very close to 2, i.e., a stoichiometry, during the entire measurement period. As expected, the rate of water splitting increases with the loading of Pt NPs as well as with Pt NP size (Table S3). The largest HE and OE rates of 25.2 and 12.9 μmol h^−1^, respectively, are obtained on the Pt107-7/TiO_2_-HA sample and the corresponding total MAs and PM-based MAs are 2521 μmol h^−1^ g^−1^ and 34.3 μmol h^−1^ mg_PM_^−1^, respectively. In a similar way, the Pt/TiO_2_-HA samples have nearly the same PM-based MA when the size of Pt NPs is the same. In contrast, the Au_50 nm_/TiO_2_-HA–Pt_25 nm_ sample exhibits a far lower activity (i.e., a HE and an OE rate of 0.54 and 0.27 μmol h^−1^, respectively, and a PM-based MA of 0.65 μmol h^−1^ mg_PM_^−1^) under visible light irradiation while the Au_50 nm_/TiO_2_-HA does not show a perceivable activity in pure water (Fig. S11b and Table S3). Also, the Pt107-7(Pt73-7)/TiO_2_-HA photocatalysts give a much higher HE/OE rate and show a larger PM-based MA that other Au-based plasmonic TiO_2_ photocatalysts in the literature (see Table S3)^29,30^. These results suggest that the plasmonic Pt NPs induce the hot electrons with more negative potentials as well as holes with more positive potentials and concomitantly serve as efficient chemical catalysts for HE and OE reactions. The Pt/TiO_2_-HA photocatalysts are robust and therefore have a high stability for catalytic water splitting. The large specific area of TiO_2_-HA and its branched features lead to a high affinity to Pt NPs so as to form a robust composite catalyst. Thus, various Pt/TiO_2_-HA samples are highly stable and do not show any degradation in the photocatalytic activity after consecutive cycles of irradiation (Fig. 3d).

Note that it is possible for our Pt NPs to catalyze the reverse reaction of water splitting, i.e., the combination of H_2_ and O_2_ into H_2_O^30^. To test the net catalytic effects of the Pt107 and Pt73 NPs, the rate of the reaction between H_2_ and O_2_ is examined in the dark using these two kinds of Pt NPs as catalysts. A very small rate of H_2_/O_2_ consumption for each catalyst is obtained, as shown in Fig. S12. This indicates that the very large Pt NPs with low surface areas is rather inactive towards the reverse reaction, hence fully contributing to water splitting.

To further corroborate the Schottky barrier height hypothesis in the photocatalytic activity enhancement of the plasmonic Pt/TiO_2_-HA in the visible region, the photoelectrochemical (PEC) properties of the Pt107-7/TiO_2_-HA and Au_50 nm_/TiO_2_-HA‒Pt_25 nm_ film electrodes are compared by conducting the photocathode half-reaction of the water-splitting process on the film electrodes and the water oxidation reaction on an un-illuminated platinum wire anode according to the method reported by Mubeen and co-workers^29^. The photocurrent density of the Pt107-7/TiO_2_-HA film electrode is considerably larger (1.8 times) than that of the Au_50 nm_/TiO_2_-HA‒Pt_25 nm_ film electrode at 0 V vs. reversible hydrogen electrode (RHE) under visible light irradiation obtained with a cut-off filter L-42 (see Fig. S13a,b). Meanwhile, electrochemical impedance spectroscopy (EIS) was conducted on these two photoelectrodes under the same illumination. A single semicircle is obtained at an applied potential of 0 V vs RHE (Fig. S13c). Concomitantly, the complex nonlinear least square (CNLS) fitting of the EIS is performed with the Zview 3.1 software package (Fig. S13c). Obviously, there is a good agreement between the experimental data (symbols) and CNLS approximations (solids lines) when the Randles equivalent circuit model is applied, as indicated by the Chi-squared value, *χ*^2^ (Table S4). The charge transfer resistance (*R*_ct_, 16.7 kΩ cm^–2^) of the Pt107-7/TiO_2_-HA photoelectrode is far lower than that (30.6 kΩ cm^–2^) of the Au_50 nm_/TiO_2_-HA‒Pt_25 nm_ photoelectrode (i.e., the faster reaction rate for the Pt107-7/TiO_2_-HA) (Table S4), consistently indicating the better photoreduction ability of plasmonic Pt hot electrons. Considering that the photocurrent density faithfully tracks the rate of hydrogen formation, the striking discrepancy in the photocurrent density between the Pt107-7/TiO_2_-HA and Au_50 nm_/TiO_2_-HA‒Pt_25 nm_ is presumably due to the fact that the higher Schottky barrier of Pt/TiO_2_-HA boosts its photoreduction ability. Concurrently, to probe the oxidation ability of holes, the Pt107-7/TiO_2_-HA and Au_50 nm_/TiO_2_-HA‒Pt_25 nm_ photoanodes are tested for PEC oxidation of water and the water reduction reaction on an un-illuminated platinum wire cathode. The obtained order of photoanode current density support the proposition that there are at least a fraction of holes photogenerated from Pt107-7/TiO_2_-HA have more positive potentials than those from Au_50 nm_/TiO_2_-HA‒Pt_25 nm_ (Fig. S13d). Similarly, the corresponding EIS data reveal that *R*_CT_ of the Pt107-7/TiO_2_-HA photoanode is much smaller than that of the Au_50 nm_/TiO_2_-HA‒Pt_25 nm_ counterpart, confirming the aforementioned conclusion (data are not shown for brevity).

**Figure 5:**
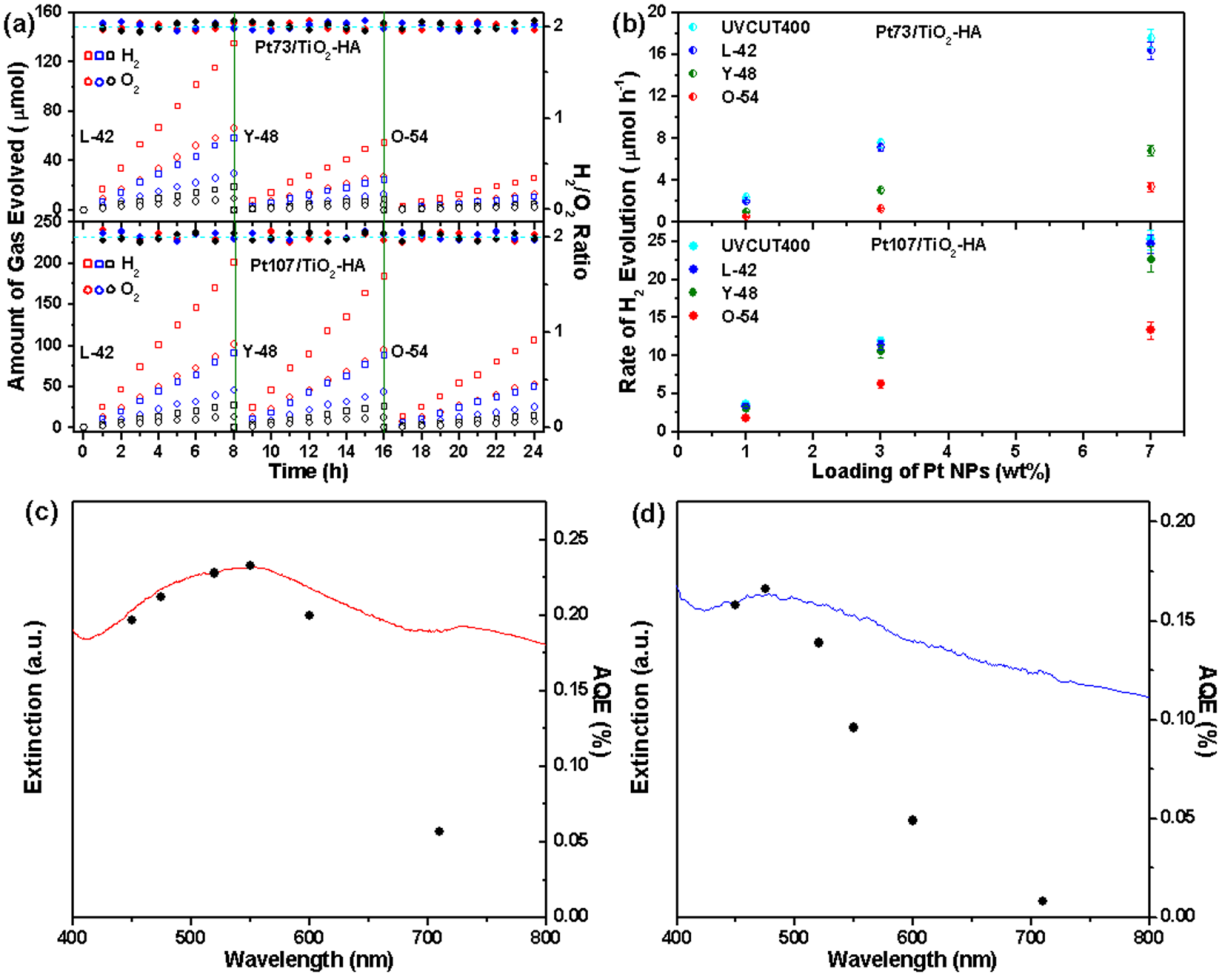
(**a**) Photocatalytic H_2_ and O_2_ evolution from pure water over time using various Pt/TiO_2_-HA (data point: red, Pt107-7 and Pt73-7; blue, Pt107-3 and Pt73-3; black, Pt107-1 and Pt73-1) photocatalysts (i.e., aqueous suspensions of various Pt/TiO_2_-HA samples) under illumination of visible light obtained from a Xe lamp equipped with different cut-off filters. (**b**) Effect of Pt loading amounts on the rate of H_2_ evolution from pure water under irradiation of visible light obtained using different cut-off filters. Extinction spectra (left axis) and action spectra (right axis) of (**c**) Pt107-7 and (d) Pt73-7 in pure water. The net extinction spectra of Pt NPs in panel (**c**) and (**d**) are obtained by subtracting of TiO_2_-HA from the corresponding extinction spectrum of Pt/TiO_2_-HA.

A more elaborate evaluation of the Pt NPs SPR-driven hot electron injection mechanism, the time course of water splitting under irradiation of visible light with longer wavelengths, which are obtained by L-42, Y-48, and O-54 cut-off filters, is shown in Fig. 5a. Evidently, the rates of HE and OE decrease with irradiation through the filter with the longer cut-off wavelength (i.e., in the order of L-42 > Y-48 > O-54), which can be attributed to the diminution of light absorption. As stated above, an increase in the loading of plasmonic Pt NPs increases both the hot electrons injected and catalytic active sites. As a result, there is an approximate linear correlation between the loading of plasmonic Pt NPs and the rates of H_2_/O_2_ formed, as shown in Fig. 5b. Figure 5c,d representatively illustrate the action spectra of the Pt107-7/TiO_2_-HA and Pt73-7/TiO_2_-HA photocatalysts, in which H_2_ formation is achieved by conducting photocatalytic tests under monochromatic irradiation of visible light. Note that compared to the intensity of corresponding extinction spectrum, the relative HE rates of these two photocatalysts obtained under red and near-IR light illumination are lower. This discrepancy can be attributed to a superimposition of both the significant light-scattering caused by the larger Pt NPs^49^ and the concurrent pronounced absorption from intraband transitions of Pt NPs^15,51^, which induce no photochemical reactions, on the broad Pt LSPR peak. Thus, the HE rate is in good accord with the plasmon absorbance spectrum of the corresponding photocatalyst. These results undoubtedly support the proposition that the VLWS on Pt/TiO_2_-HA photocatalysts stems from the hot electrons injection excited by the SPR of the Pt NPs on TiO_2_-HA.

Apparent quantum efficiency (AQE) is an important parameter to evaluate the photocatalytic activity of a photocatalyst and is calculated in terms of the following equation:

1 $${\rm{AQE}}=\frac{{\rm{2}}\times (\mathrm{number}\,{\rm{of}}\,{{\rm{H}}}_{{\rm{2}}}\,\mathrm{molecules})}{{\rm{number}}\,{\rm{of}}\,{\rm{incident}}\,{\rm{photons}}}\times 100$$

The spectrally averaged photon energy (the visible portion (λ > 420 nm)) is *ca*. 3.98 × 10^−19^ J^29^. In our case, the amount of photons irradiated is measured to be 0.5 μmol cm^−2^ s^−1^, where the corresponding illumination intensity is 120 mW cm^−2^. The average AQEs of the Pt107-7/TiO_2_-HA and Pt73-7/TiO_2_-HA photocatalysts are calculated to be 0.18% and 0.12%, respectively. The AQE values are much larger than the value (0.013%) in ref.^29^ and comparable to the value (0.1%) in ref.^28^. However, it is believed that the value (0.1%) in ref.^28^ is questionable considering the extremely low rate of H_2_ evolution (0.25 ± 0.05 μmol h^−1^) and higher total illumination intensity (300 mW cm^−2^). Furthermore, the AQEs reach 0.23% at 550 nm and 0.17% at 475 nm for the Pt107-7/TiO_2_-HA and Pt73-7/TiO_2_-HA, respectively (Fig. 4c,d). Clearly, the rather broad SPR absorption of Pt NPs leads to the moderate increase in the AQE around the maximum in the plasmon spectrum in comparison with the corresponding average AQE. In addition, as anticipated, the AQE of the Pt107-7/TiO_2_-HA is more than one order of magnitude higher than that of the Au_50 nm_/TiO_2_-HA‒Pt_25 nm_ at the corresponding wavelength (Fig. S11c). However, it is not surprising that the AQE of the Pt107-7/TiO_2_-HA is still too low, considering that the photocatalyst mainbody TiO_2_-HA does not absorb visible light at all. Thus, usage of modified TiO_2_-HA with significant visible-light absorption ability will be an effective pathway to further improve the AQE.

To further evaluate the long-term stability and durability of Pt/TiO_2_-HA, the Pt107-7/TiO_2_-HA and Pt73-7/TiO_2_-HA photocatalysts are tested for 8 h each day and then used again by centrifugation of their aqueous suspension. The process is continually repeated for a week. The photocatalysts do not show any perceptible degradation in photocatalytic activity after one week of operation because of the highly robust nano-architecture of the Pt/TiO_2_-HA photocatalysts as well as the exceptional chemical stability and anti-photocorrosion ability of Pt and TiO_2_ (see Fig. S14).

## Conclusions

In summary, the Pt/TiO_2_-HA heterostructures are successfully fabricated and employed as efficient photocatalysts for plasmon-enhanced visible light hydrogen production. The plasmonic Pt NPs not only effectively activate the photocatalytic activity of pure TiO_2_ in the visible region but also eliminate the use of additional chemical co-catalysts for HE and OE reactions in the VLWS process. Hence, the Pt/TiO_2_-HA heterostructure is expected to behave as an emerging powerful photocatalyst with enhanced photocatalytic activity and a simple composition for sustainable development of solar energy conversion. Our studies pave the way for the development of efficient new plasmonic photocatalysts by extending and optimizing plasmonic species coupled with tailoring unique nanostructures. At the same, it is expected that the plasmonic Pt nanostructures can be extended to more types of semiconductor supports, such as α-Fe_2_O_3_, for further optimizing the performance of plasmonic photocatalysts to drive the unassisted full water splitting reaction.

## Methods

### Chemicals

Potassium titanium oxide oxalate dihydrate (K_2_TiO(C_2_O_4_)_2_·2H_2_O, ≥98% Ti basis), hydrogen hexachloroplatinate(IV) hexahydrate (H_2_PtCl_6_·6H_2_O, 99.9%), sodium borohydrate (NaBH_4_, 98%), sodium hypophosphite monohydrate (NaH_2_PO_2_·H_2_O, 99%), diethylene glycol (99%), and urea (H_2_NCONH_2_, 99%) were commercially available from Sinopharm Chemical Reagent Co., Ltd. Hexadecyltrimethylammonium bromide (CTAB, >99%), _L_-ascorbic acid (_L_-AA, >99%), sodium citrate tribasic dihydrate (99%), and citric acid (99.5%) were purchased from Sigma-Aldrich. All reagents were used without any further purification. Ultrapure water (18.2 MΩ) produced with a Milli-Q purification system was used in the synthesis and photocatalytic measurements.

### Synthesis of TiO_2_-HA

Typically, K_2_TiO(C_2_O_4_)_2_·2H_2_O (156 mg) and NaH_2_PO_2_·H_2_O (42.4 mg) were added to a mixed solvent of water (5.0 mL) and diethylene glycol (15.0 mL), and then sonicated for 10 min. Subsequently, the reaction mixture was loaded into a 40 mL Teflon-lined stainless steel autoclave and vigorously stirred by a magnetic stirrer at ambient temperature for 30 min. Next, the autoclave was sealed and maintained at 180 °C for 12 h and subsequently cooled to room temperature naturally. Finally, the white sediments at the bottom of the autoclave were isolated by centrifugation of the resulting mixture at 2000 rpm for 5 min followed by washing with hot water and ethanol (80 °C) alternatively for three times to purify the final product. The purified sediments were dried at 100 °C overnight to obtain the anatase-structured TiO_2_-HA for further characterization and preparing photocatalysts.

### Synthesis of plasmonic Pt NPs and Pt/TiO_2_-HA composites

For comparison, large plasmonic Pt colloidal NPs with a mean diameter of *ca*. 73 or 107 nm, which show maximum extinction wavelengths of 455 or 542 nm, respectively, were synthesized according to the procedure reported by Bigall *et al*.^35^. Unlike the multi-step process described by Bigall *et al*.^35^, we develop a one-step approach to achieve the composites of large plasmonic Pt NPs-decorated TiO_2_-HA (Pt/TiO_2_-HA). First, 5 nm Pt seeds were prepared using the procedure according to Bigall *et al*.^35^. Briefly, an aqueous solution of H_2_PtCl_6_·6H_2_O (36 mL, 0.2%) was added to 464 mL of boiling water. After one min, a solution of sodium citrate (1%) and citric acid (0.05%) was added to the above H_2_PtCl_6_ solution, and 0.5 min later a freshly prepared NaBH_4_ solution (5.5 mL, 0.08%) containing sodium citrate (1%) and citric acid (0.05%) was rapidly injected into the above reaction solution. After reaction for 10 min, the Pt seed solution was cooled down to room temperature. To obtain the Pt/TiO_2_-HA with a varied Pt loading (i.e., 1 wt%, 3 wt%, or 7 wt% Pt), a different amount of TiO_2_-HA powder (1110, 370, or 158.6 mg) was dispersed in 27 mL of water in a three-necked flask (fitted with a magnetic stirrer, a reflux condenser, and a thermometer) under vigorous stirring for 30 min. Afterwards, small 5 nm Pt seeds in 0.25 (for the Pt107/TiO_2_-HA) or 0.60 mL (for the Pt73/TiO_2_-HA) of water was added to the TiO_2_-HA suspension and fully mixed with each other under vigorous stirring for another 30 min. Next, to the above mixture, 0.45 mL of an aqueous H_2_PtCl_6_ solution (0.20 M) and 2.5 mL of an aqueous solution containing sodium citrate (1.0 wt%) and _L_-ascorbic acid (1.25 wt%) were added. The resultant reaction mixture was heated to the boiling point (~100 °C) at a rate of ~8 °C min^−1^ and then refluxed at this temperature for 30 min. After the mixture was cooled to ambient temperature, the supernant of the suspension became colorless and the pristine white TiO_2_-HA powder turned into light grey or grey and precipitated at the bottom of the flask, indicating the complete attachment of Pt NPs onto the TiO_2_-HA. Finally, the resulting Pt/TiO_2_-HA precipitates were isolated by centrifugation and thoroughly washed with hot water (50 °C) three times to remove the adsorbed organic species and then were dried in a vacuum oven at 80 °C for 12 h. Note that Pt NPs show a strong adhesion to the surface of TiO_2_-HA with ample spacings among b-NWs and very high surface energy so as to allow for the direct contact between Pt NPs and the TiO_2_-HA surface after cleaning with water, thus enabling a higher photocatalytic activity. The final Pt/TiO_2_-HA samples were further treated by oxygen plasma etching to completely remove the organic residues.

### Characterization of materials

Scanning electron microscopy (SEM) was performed using a Hitachi S-4800 field-emission scanning electron microscope operating at 5 kV to investigate the morphology and nano/micro-structure of the samples. Transmission electron microscopy (TEM) micrographs were obtained using a FEI Tecnai G2 Spirit Bio TWIN transmission electron microscope operating at an accelerating voltage of 120 kV. Specimens for TEM observations were sonicated before dropping them onto 300 mesh carbon-coated copper grids. High resolution TEM (HRTEM) micrographs, high-angle annular dark field (HAADF) scanning transmission electron microscopy (STEM) images, and energy-dispersive X-ray spectroscopy (EDS) elemental maps were acquired using a FEI Tecnai G2 F20 S-Twin electron microscope operating at 200 kV. X-ray photoelectron spectroscopy (XPS) measurements were carried out using a PHI5000 VersaProbe (ULVAC-PHI) spectrometer with an energy analyzer, employing a monochromatized microfocused Al Kα (*hv* = 1,486.58 eV) X-ray source. Samples for XPS measurements were pretreated by repeated cycles of Ar+ ion sputtering to obtain clean sample surfaces. The binding energies (BEs) of the core levels were calibrated by setting the adventitious C 1 s peak at 284.8 eV. Survey spectra of the samples in the BE range of 0–1,000 eV, and the core level spectra of the elemental signals were recorded at resolutions of 1 and 0.125 eV, respectively. The X-ray diffraction (XRD) patterns were recorded using a Rigaku SmartLab diffractometer with Cu Kα radiation (λ = 1.5406 Å) operating at 40 kV and 100 mA at a scanning rate of 0.06°·s^−1^. N_2_ adsorption-desorption isotherm analysis was conducted at 77 K using a BELSORP-max micropore analyzer. The Brunauer–Emmett–Teller (BET) specific surface area (SSA) and the pore size distribution (PSD) of the TiO_2_-HA sample were obtained based on N_2_ adsorption isotherms in the relative pressure (*P*/*P*_0_) range from 0.04 to 0.50 and non-local density functional theory (NLDFT) calculations by using nitrogen adsorption data and assuming a slit pore model, respectively. The samples were degassed under high vacuum (<0.01 mbar) at 200 °C for at least 6 h prior to the measurements. UV-vis extinction spectra were recorded using a Shimadzu UV-3600 UV–vis–NIR spectrophotometer equipped with a LISR-3100 150 mm integrating sphere. The diffuse reflection spectra of all the samples were obtained using BaSO_4_ as a standard reference in the measurements.

### Photocatalytic measurements

To assess the photocatalysis performance of various Pt/TiO_2_-HA samples, hydrogen production from pure water was monitored in the presence or absence of ethanol. In the case of using ethanol as the model sacrificial agent, each Pt/TiO_2_-HA sample (10 mg) was suspended in an aqueous solution of ethanol (100 mL, the volume ratio of water to ethanol is 4: 1) in a closed-gas circulation reactor. For overall water splitting, a 10 mg sample of Pt/TiO_2_-HA powder was dispersed in 80 mL of pure water. The reactant suspension was evacuated under vacuum three times and then an argon flow (12.5 mL min^−1^) was introduced into the reaction system to completely remove air from the reactor. Concomitantly, the argon flow also served as the carrier gas to carry the reaction products to the detector. Afterwards, the suspension was irradiated using a 300 W xenon lamp (HSX–UV300) equipped with various cut-off filters (UVCUT400, L-42, Y-48, and O-54) for irradiating the photocatalysts with different illumination wavelengths. The amounts of gaseous products (H_2_) was analyzed by a gas chromatograph ((GC, Shimadzu, GC-8A) using a thermal conductivity detector (TCD).

### Finite-difference time-domain (FDTD) analysis

The enhancement of electric field at the interface of Pt107/TiO_2_-HA and Au_50 nm_/TiO_2_-HA was calculated by using a software package, FDTD Solutions 8.15 (Lumerical Solutions, Inc.). During simulations, an electromagnetic pulse in the wavelength range from 400 to 700 nm (for Pt107/TiO_2_-HA) or 450 to 700 nm (for Au_50 nm_/TiO_2_-HA) was launched into a box containing a target nanostructure. A size of 1 × 1 × 1 nm^3^ was chosen for the override mesh cell. The model was set up by using a Pt nanosphere of 107 nm in diameter supported on a TiO_2_ nanoring of 250 nm in outer diameter and 100 nm in inner diameter. The optical constants of Pt and Au were adopted from tabulated values of bulk platinum and gold (Palik), respectively, while that of TiO_2_ (anatase) as adopted from Hand book of Optical Constants of Solids^53^.

## Electronic supplementary material

Supplementary Information
