# Supplementary material for: Plasmonic Pt nanoparticles—TiO2 hierarchical nano-architecture as a visible light photocatalyst for water splitting
Source: Sci Rep. 2018 Nov 1;8:16198. doi: 10.1038/s41598-018-33795-z (PMC6212491; doi:10.1038/s41598-018-33795-z)
Supplement: Supplementary file 1 — Supplementary Information [file 41598_2018_33795_MOESM1_ESM.docx]

**Supplementary Information**

**Plasmonic Pt Nanoparticles—TiO_2_ Hierarchical Nano-architecture as a Visible Light Photocatalyst for Water Splitting**

**Lipei Qin, Guojing Wang, and Yiwei Tan***

*State Key Laboratory of Materials-Oriented Chemical Engineering, School of Chemistry and Chemical Engineering, Nanjing Tech University, Nanjing 210009, China, Email:* [*ytan@njtech.edu.cn*](mailto:ytan@njtech.edu.cn)*, Tel: +86-25-83172267*

**Experimental Section**

**Synthesis of 50 nm Au Nanoparticles (NPs) Supported on TiO_2_-HA with 25 nm Pt NP Co-catalyst (Au_50 nm_/TiO_2_-HA–Pt_25 nm_).**

(i) *Synthesis of Small ~3 nm Au Seeds*. Typically, 0.62 mL of an aqueous solution of HAuCl_4_ (47.63 mM) was added to 90.0 mL of water under vigorous magnetic stirring. After 1 min of stirring, to the above solution, 2.0 mL of sodium citrate (38.8 mM) was added, and then the mixture was vigorously stirred for 1 min at ambient temperature. Subsequently, 1 mL of an aqueous solution containing NaBH_4_ (0.075 wt%) and sodium citrate (38.8 mM) was added to above mixture, followed by 5 min of vigorous stirring. The resulting Au colloids were stored at 4 ºC.

(ii) *Synthesis of Au_50 nm_/TiO_2_-HA*. 0.62 mL of an aqueous solution of HAuCl_4_ (47.63 mM) was added to 49.0 mL of water. Afterward, TiO_2_-HA powder (69 mg) was suspended in the above solution under vigorous magnetic stirring, and then the suspension was heated to the boiling point (~100 ºC) at a rate of ~5 ºC min^–1^. Next, 40 μL of Au seed solution and 0.44 mL of an aqueous solution of sodium citrate (38.8 mM) were concurrently added to the above boiling solution, followed by heating at 100 ºC for 30 min. After the reaction mixture was cooled to ambient temperature, the supernant of the suspension became colorless and the pristine white TiO_2_-HA powder turned into grey and precipitated at the bottom of flask, indicating the complete attachment of Au NPs onto the TiO_2_-HA. Finally, the resulting Au_50 nm_/TiO_2_-HA precipitates were separated by centrifugation and washed with hot water (50 ºC) three times to remove the adsorbed organic species, and then were dried in a vacuum oven at 80 ºC for 12 h.

(iii) *Synthesis of Au_50 nm_/TiO_2_-HA–Pt_25 nm_*. The as-obtained Au_50 nm_/TiO_2_-HA powder (50 mg) was suspended in 150.0 mL of water. After the suspension was vigorously stirred for 30 min, 1.80 mL of 5 nm Pt seeds was added, followed by stirring for another 30 min. Then, to the suspension, 400 μL of an aqueous solution of H_2_PtCl_6_ (0.04 M) and 500 μL of an aqueous solution containing sodium citrate (1.0 wt%) and _L_-ascorbic acid (1.25 wt%) were added in order. Afterward, the reaction system was heated to the boiling point (~100 ºC) at a rate of ~5 ºC min^–1^ and then refluxed at this temperature for 30 min. After the mixture was cooled to ambient temperature, the resulting Au_50 nm_/TiO_2_-HA–Pt_25 nm_ precipitates were isolated by centrifugation and thoroughly washed with hot water (50 ºC) three times to remove the adsorbed organic species and then were dried in a vacuum oven at 80 ºC for 12 h.

**Fabrication of the Pt107-7/TiO_2_-HA and Au_50 nm_/TiO_2_-HA‒Pt_25 nm_ Film Electrodes.**

A uniform aqueous suspension of TiO_2_-HA (0.5 mg mL^‒1^) was prepared by vigorous sonication, and then deposited onto a piece of clean fluorine-doped tin oxide coated glass (FTO, 10‒13 Ω cm^‒2^, 0.5 cm by 2.0 cm), which is used as a transparent conducting electrode, by dropcasting via very slow solvent evaporation. During the process, doctor blading was applied to coat the suspension onto the horizontal FTO substrate (achieving coverage over an area of 0.5 cm by 1.5 cm) to obtain a uniform thickness and loading of TiO_2_-HA (0.35 mg cm^‒2^). After complete evaporation of water, thermal annealing of the TiO_2_-HA films on FTO was performed in a tube furnace under an air atmosphere at 500 °C for 2 h, leading to the formation of a strongly adherent TiO_2_-HA film on FTO substrate. The TiO_2_-HA/FTO electrode with the back side coated with an adhesive and easily peeled clean tape was immersed into 27 mL of an aqueous suspension of TiO_2_-HA powder (158.3 mg). Next, the Pt107-7/TiO_2_-HA film electrodes were prepared by the same procedure for synthesizing the plasmonic Pt107-7/TiO_2_-HA composite and then withdrawn from the resulting reaction mixture and cleaned using the same procedure as that for the synthesis of Pt/TiO_2_-HA composites (see above). Similarly, for preparing Au_50 nm_/TiO_2_-HA‒Pt_25 nm_ film electrodes, the preceding TiO_2_-HA/FTO electrode was immersed into 49.62 mL of an aqueous TiO_2_-HA (68.7 mg) suspension containing HAuCl_4_ (0.595 mM). Afterwards, the Au_50 nm_/TiO_2_-HA film electrode was fabricated and cleaned following the same procedure for synthesizing the Au_50 nm_/TiO_2_-HA composite as described above. Finally, the Au_50 nm_/TiO_2_-HA film electrode was immersed into 150.0 mL of an aqueous suspension of Au_50 nm_/TiO_2_-HA powder (49.7 mg). Following the same procedure as that for synthesizing the Au_50 nm_/TiO_2_-HA–Pt_25 nm_ powder, we have achieved the fabrication of Au_50 nm_/TiO_2_-HA‒Pt_25 nm_ film electrodes.

**Photoelectrochemical (PEC) Measurements.** PEC measurements were conducted in a three-electrode PEC cell with a platinum wire as the counter electrode in the dark and Ag/AgCl as the reference. To prepare the working electrode (photocathode), a copper wire was connected to the bare upper part of the FTO substrate of the Pt107-7/TiO_2_-HA or Au_50 nm_/TiO_2_-HA‒Pt_25 nm_ film electrode. There is a spacing of about 1 mm between the film edge and the metal contact. The working electrode area is 0.75 (0.5 × 1.5) cm^2^. A Na_2_SO_4_ (1 M) aqueous solution buffered at pH 5 or 10 was used as an electrolyte for PEC measurements. The working electrode and the counter electrode are separately placed in a compartment separated by a glass frit containing nafion dispersions, allowing only protons to pass through. All linear scan voltammetry curves (i.e., photocurrent density curves) were recorded at a scan rate of 5 mV s^‒1^ under illumination or in the dark by a CHI 660D electrochemical potentiostat. A 300 W xenon lamp (HSX–UV300) equipped with a cut-off filter L-42 was used as light source for irradiating only the Pt107-7/TiO_2_-HA or Au_50 nm_/TiO_2_-HA‒Pt_25 nm_ film photoelectrode. The intensity of the incident light near the photocathodes (120 mW cm^‒2^) was measured using a photometer. Electrochemical impedance spectroscopy (EIS) spectra were recorded using the same potentiostat in the preceding three-electrode configuration. A sinusoidal voltage perturbation, with amplitude of 5 mV and frequencies ranging from 10^5^ to 1 Hz, was superimposed on the bias voltage to gather the EIS data under the same illumination. The impedance was measured at bias voltage of 0 or 1.23 V vs reversible hydrogen electrode (RHE) for the photocathode or photoanode, respectively. Meanwhile, the EIS data were fit to the equivalent circuits, as discussed in the following using the CHI 660D software.

**Figure S1.** XPS spectra of the TiO_2_-HA sample without washing pretreatment. (a) XPS survey spectrum. (b) Ti 2p, (c) O 1s, and (d) P 2p detail spectra.


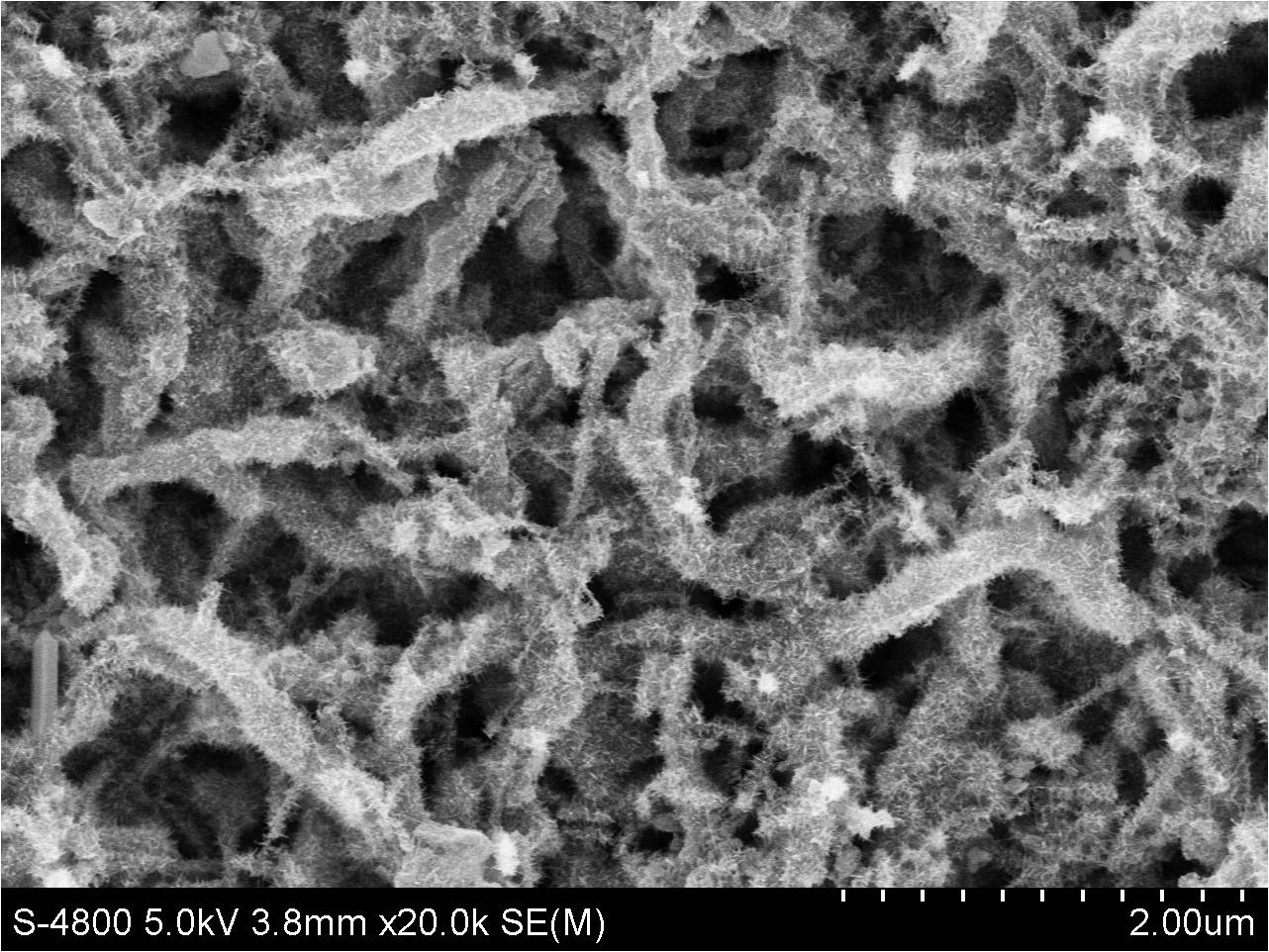

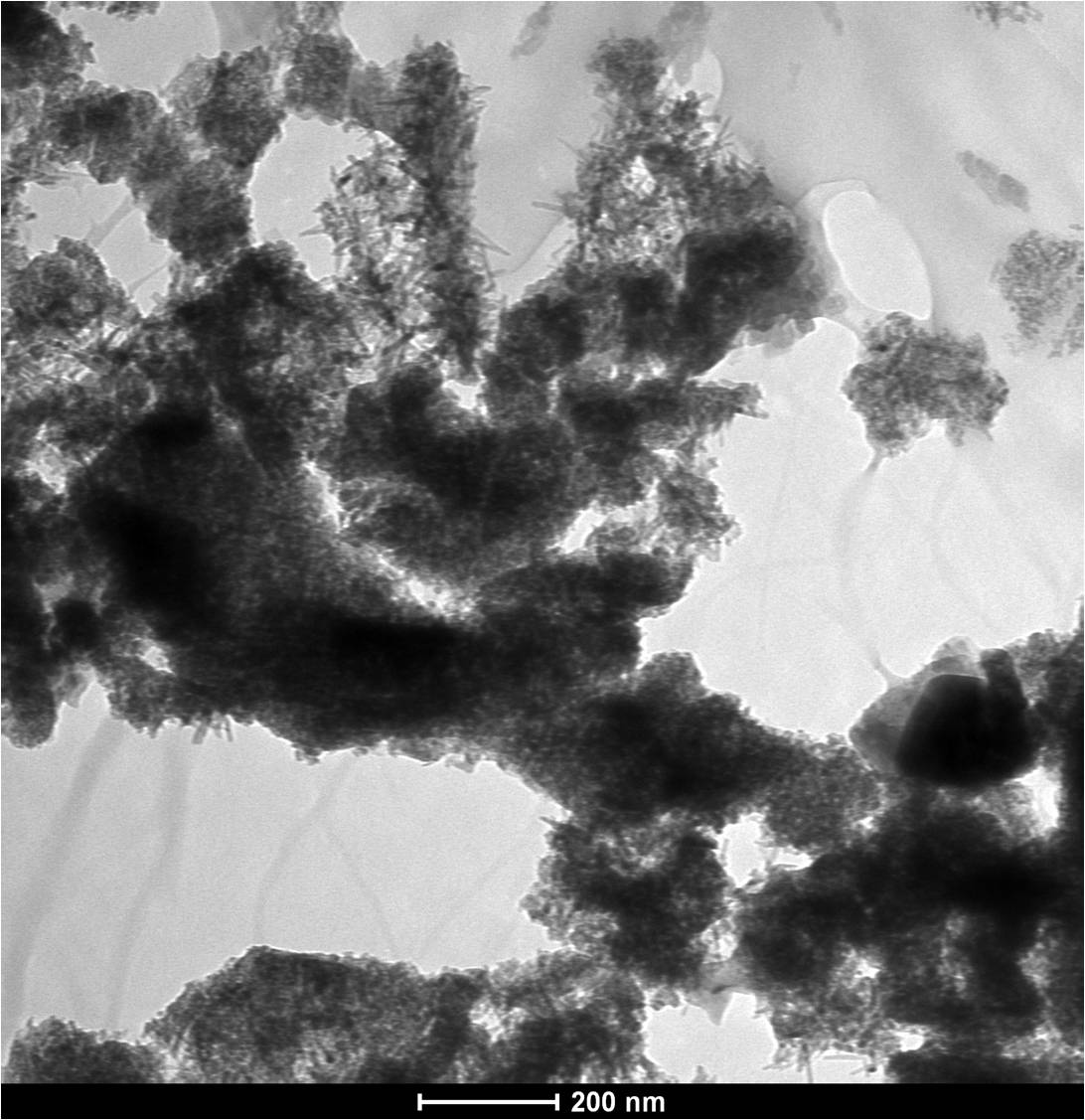


**Figure S2.** (a) SEM and (b) TEM images of the nonuniform TiO_2_ branched structures obtained in the absence of NaH_2_PO_2_ under otherwise the same experimental conditions as those for preparing the TiO_2_-HA.

The survey spectrum reveals the presence of Ti and O elements besides the adventitious carbon species (Figure S1a). The XPS high resolution spectrum of Ti 2p in Figure S1b shows that two BEs centered at 458.1 and 464.1 eV can be assigned to Ti 2p_3/2_ and Ti 2p_1/2_, respectively. The positions of the Ti 2p_1/2_ and Ti 2p_3/2_ peaks are consistent with those of completely oxidized Ti(IV) species.^1^ The O 1s spectrum is fitted into two peaks centered at 529.5 and 531.4 eV by deconvolution (Figure S1c), which can be tentatively assigned to the oxygen species associated with Ti(IV)–O and adsorbed hydroxyl group (O–H), respectively.^2^ The surface chemical composition of the TiO_2_-HA is determined to be a Ti : O atomic ratio of 29.85 : 70.15, which is in good accord with the bulk Ti : O atomic ratio of 33.02 : 66.98 obtained by EDS quantitative analysis.

XPS measurements also reveal the presence of trace surface P species (133.0 eV, P‒O species of phosphate) on the surface of the TiO_2_-HA sample without pretreatment by washing (Figure S1d).^3,4^ This can be ascribed to the adsorption and oxidation of hypophosphite during the solvothermal growth of TiO_2_-HA, clearly underlining that the additive (H_2_PO_2_^‒^) plays a pivotal role in controlling the structure of TiO_2_-HA. It should be pointed out that no changes occur in the bandgap and the conductivity of TiO_2_-HA even though P‒O species (in our case, the inert K_3_PO_4_ or Na_3_PO_4_) are attached onto TiO_2_-HA based on the previous studies.^5,6^ To probe the effects of H_2_PO_2_^‒^ on the growth of TiO_2_ under our solvothermal conditions, we conducted a control experiment on the synthesis of TiO_2_ in the absence of NaH_2_PO_2_ while keeping other experimental parameters unchanged. Both SEM and TEM images reveal that the product has a noticeably different morphology and structure, as shown in Figure S2a and S2b, respectively. It can be seen that the ill-defined TiO_2_ branched structures with non-uniform sizes and irregular morphologies are produced. The disproportionation of hypophosphite anions is known to occur at high temperature in solution to yield PH_3_ tiny bubblets which can template hollow superstructures, giving rise to the formation of the TiO_2_-NAs with a tubular primary structure. On the other hand, a portion of H_2_PO_2_^‒^ ions that act as ligands might react directly with Ti(IV) ions to generate Ti‒OPOH_2_. It is expected that the formation of Ti‒OPOH_2_ is helpful for adjusting the polymerization rate to form TiO_2_ nano-branches, leading to a more homogeneous degree of TiO_2_ branching growth. Similarly, in a very recent report, Chen et al. demonstrated the synthesis of hollow spherical TiO_2_ nanostructures using sodium hypophosphite as a structure-directing agent under hydrothermal conditions.^7^ Evidently, NaH_2_PO_2_ plays a pivotal role in inducing growing hollow tubular primary structure as well as achieving more uniform branching. However, the more detailed and accurate mechanisms for the formation of TiO_2_-NA need to be further explored and are beyond the scope of this manuscript. The adsorbed P species can be removed by thoroughly washing with hot water and ethanol (80 ºC) in combination with oxygen plasma etching, as evidenced by the EDS spectrum in Figure S4c (see the discussion below) and the high resolution P 2p XPS spectrum in Figure S5e.

**Figure S3.** (a) N_2_ (77.4 K) adsorption–desorption isotherm and (b) pore size distribution (PSD) of the TiO_2_-HA, which is calculated from the data using a slit/cylindrical NLDFT model. The inset in panel (b) shows the PSD in the range of 0–30 nm.

The occurrence of the dramatic adsorption hysteresis observed in Figure S3a is invoked by capillary condensation in overwhelming majority of mesopores and/or macropores. The PSD shown in Figure S3b reveals a dominant peak of macropores at *ca*. 135 nm, a weak peak of mesopores centered at *ca*. 11 nm (formed by the interspacing among b-NWs), and small peaks of nanopores with size smaller than 4 nm. These results further verify the formation of hollow tubular structure.


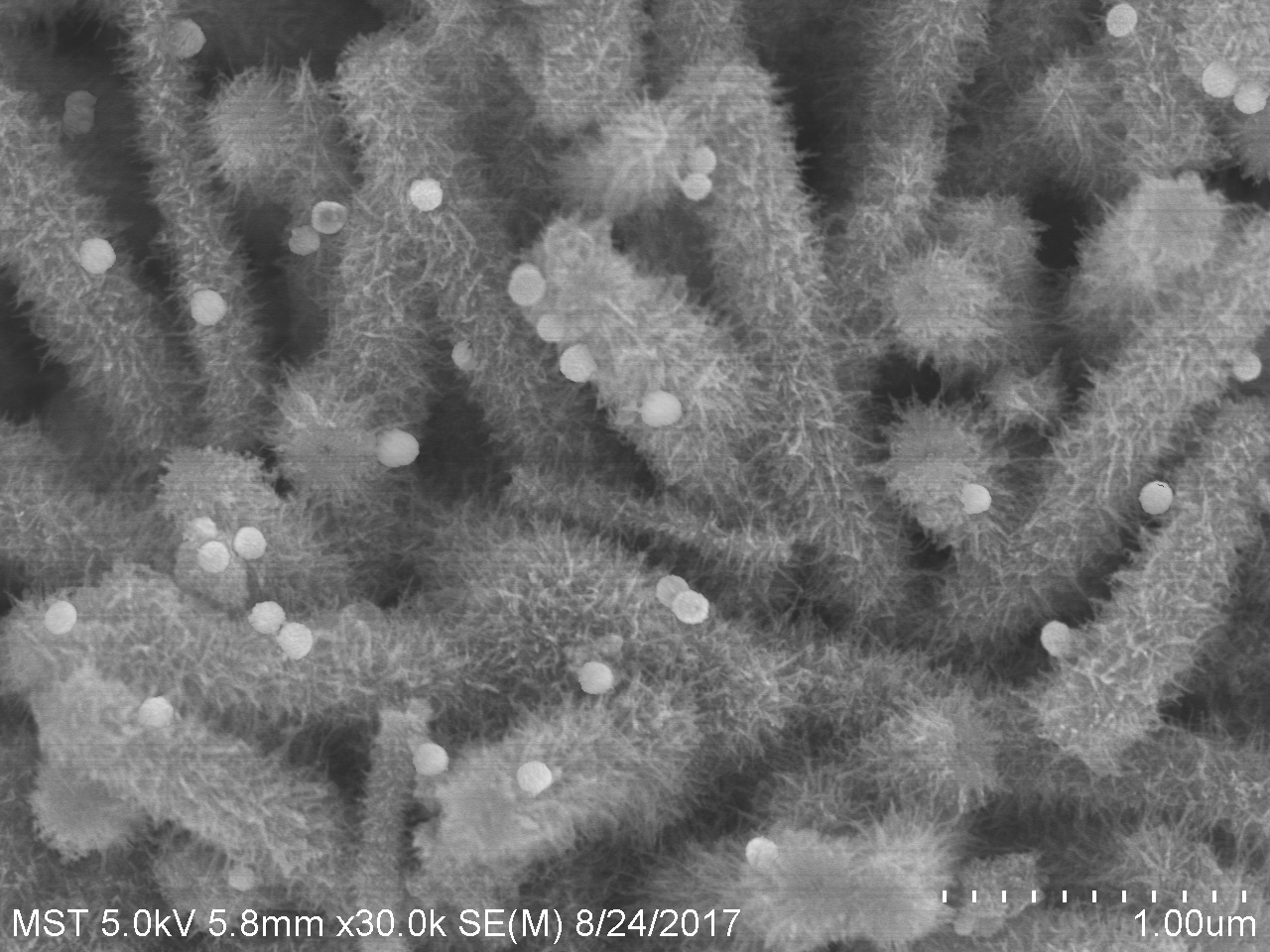

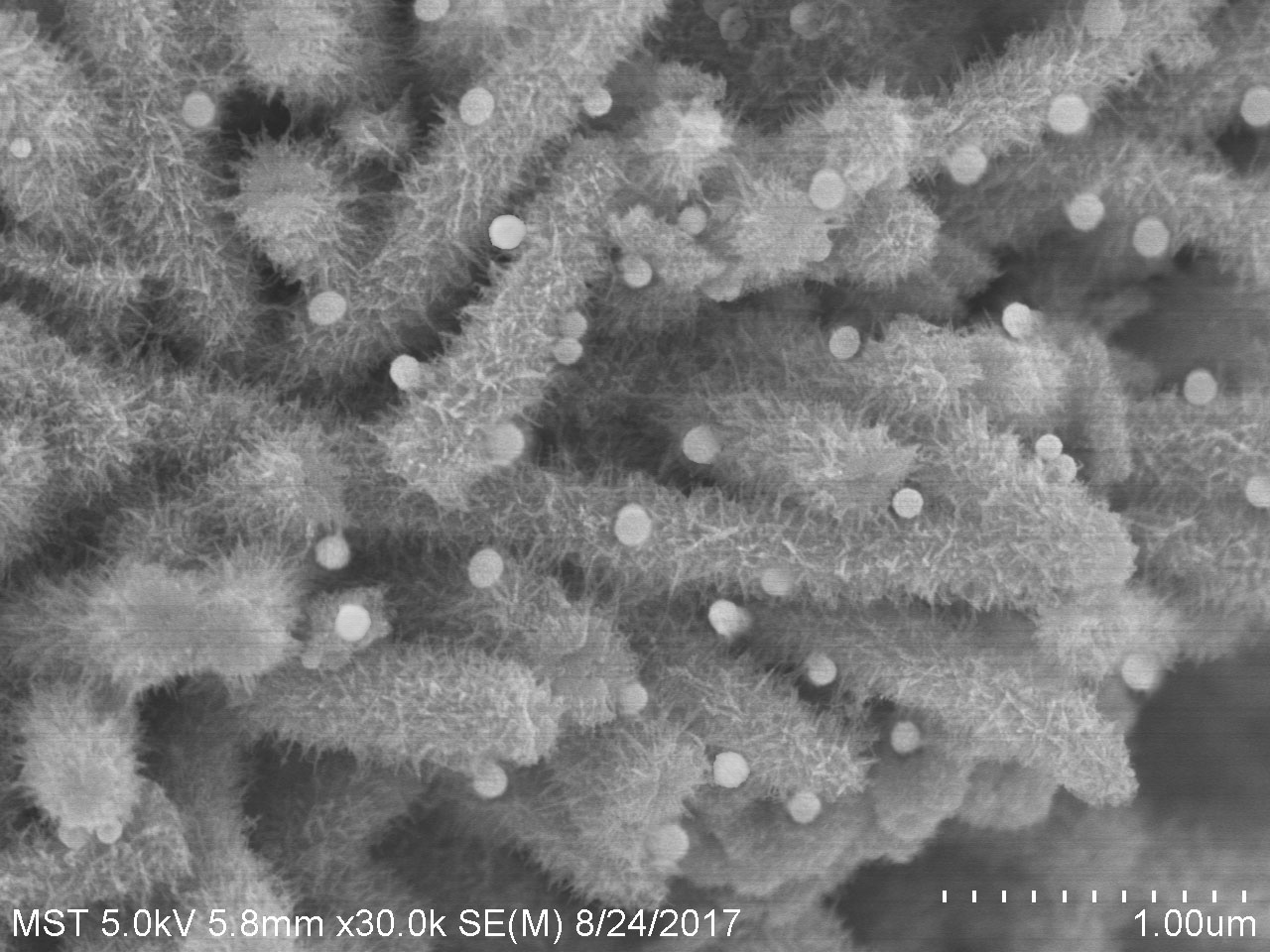

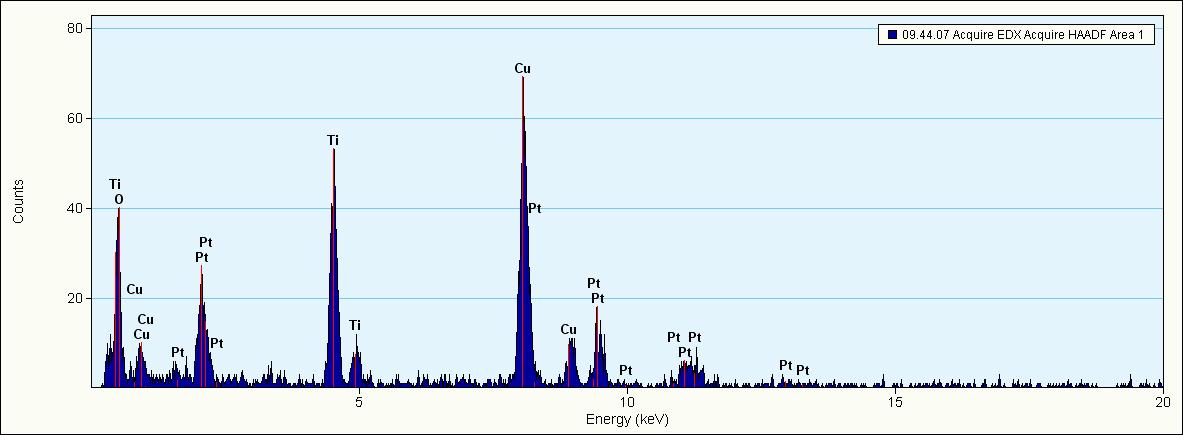


**Figure S4.** SEM images of (a) the Pt107-1/TiO_2_-HA and (b) Pt107-3/TiO_2_-HA containing 1 wt% and 3 wt% 107 nm Pt NPs, respectively. (c) EDS spectrum of the Pt107-7/TiO_2_-HA sample, in which the Cu signal originates from the copper grid used for TEM imaging. No P signals can be found, indicating the phosphorus content both in the bulk and on the surface of TiO_2_-HA are negligible. The corresponding size distribution histograms for the TiO_2_-HA-supported Pt NPs with an average diameter of 73 and 107 nm are shown in panel (d) and (e), respectively, which are obtained by counting more 300 individual NPs found in several arbitrarily chosen areas in SEM images.

**Figure S5.** XPS spectra of the Pt107-7/TiO_2_-HA. (a) XPS survey spectrum. (b) Ti 2p, (c) O 1s, (d) Pt 4f, and (e) P 2p core level spectra.

**Figure S6.** UV-vis extinction spectra of the Pt73-1/TiO_2_-HA, Pt73-3/TiO_2_-HA, Pt107-1/TiO_2_-HA, and Pt107-3/TiO_2_-HA samples.

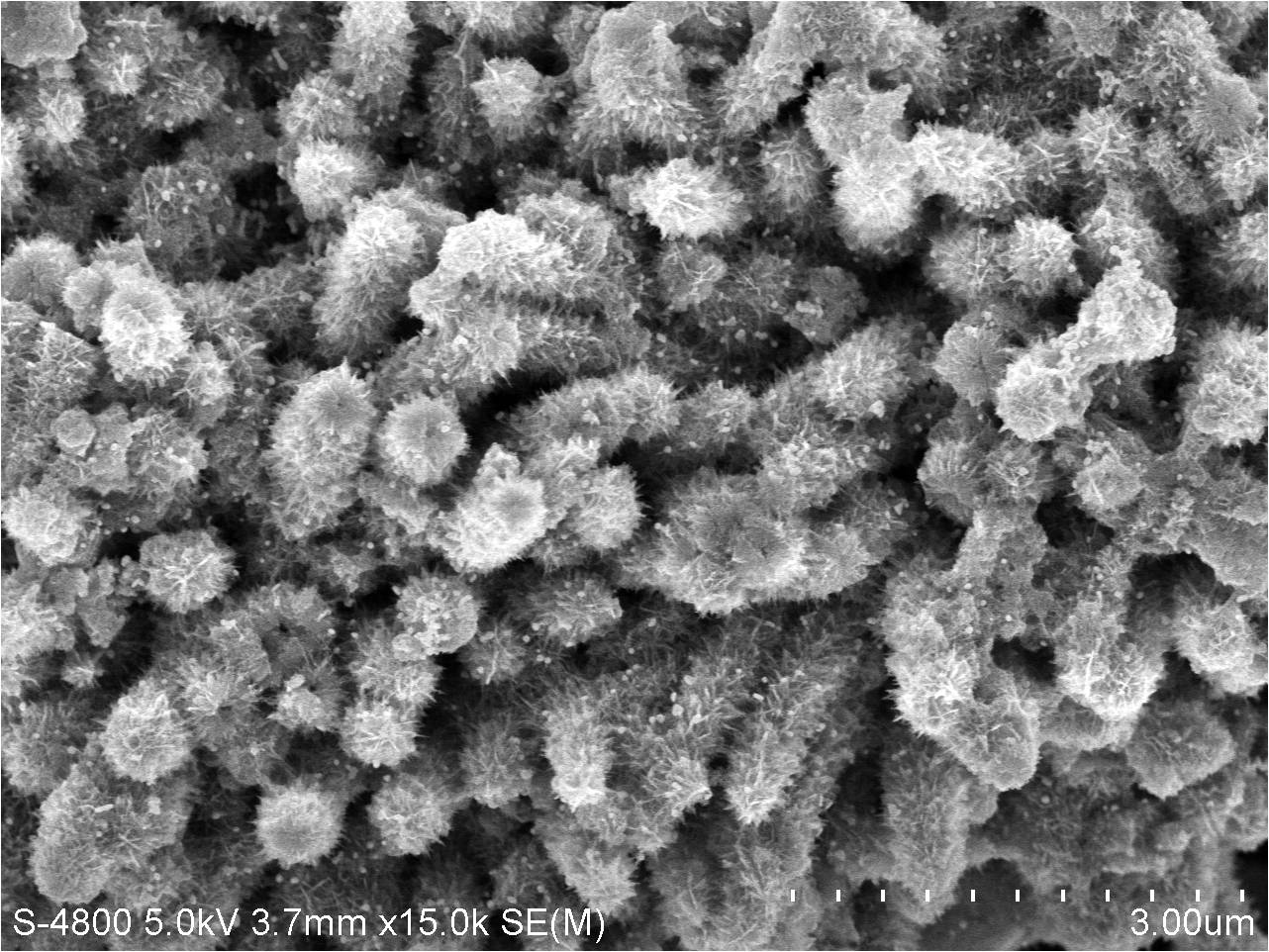


**Figure S7.** (a) SEM image and (b) time course of evolved H_2_ under visible light (*λ* > 400 nm) irradiation of the TiO_2_-HA decorated with ~25 nm Pt NPs, which was prepared using TiO_2_-HA powder (50 mg) as the precursor while keeping other conditions the same as the synthesis of Au_50 nm_/TiO_2_-HA–Pt_25 nm_.

**Figure S8.** (a) Open circuit voltage (*V*_OC_)–time profiles under light on and off conditions and (b) average electron lifetime of the Pt107-7/TiO_2_-HA and the Au_50 nm_/TiO_2_-HA and TiO_2_-HA control samples.

The *V*_OC_–time profile is recorded using a three-electrode PEC cell as described in the Experimental Section to probe the hot electron dynamics within the TiO_2_-HA CB by exciting the SPR of the Pt107-7/TiO_2_-HA under λ > 420 nm irradiation and by comparing it with the corresponding profiles from the Au_50 nm_/TiO_2_-HA (the details of its structures are discussed below) and TiO_2_-HA control samples (see Figure S8a). Similar to the previous report by DuChene et al.,^8^ upon light on, the *V*_OC_ decreases for all the photoelectrodes, arising from the accumulation of hot electrons within the TiO_2_-HA conduction band (CB) for the Pt107-7/TiO_2_-HA and Au_50 nm_/TiO_2_-HA or intrinsic bandgap transition for TiO_2_-HA. At the same time, compared to TiO_2_-HA, the gradual descending ramp of *V*_OC_ is observed at the initial stage of irradiation, being indicative of the limited transmission probability across the plasmonic metal–interface. In particular, the Pt107-7/TiO_2_-HA exhibits a greater drop of *V*_OC_ than the Au_50 nm_/TiO_2_-HA, presumably due to the more efficient injection of hot electrons into the TiO_2_-HA CB under excitation with the shorter visible light. Upon ceasing the illumination, the decay rate of *V*_OC_ follows the order of Pt107-7/TiO_2_-HA < Au_50 nm_/TiO_2_-HA < TiO_2_-HA. Furthermore, the average lifetime of the photogenerated carriers (τ_n_) is plotted against *V*_OC_ in Figure S8b according to the following formula:^8^

where τ_n_ is the average electron lifetime, *k*_B_, *T*, and *q* are the Boltzmann constant, temperature, and charge of an electron, respectively. As can be seen, the hot electrons in the Pt107-7/TiO_2_-HA exhibit a longer lifetime of (ca. 3500 s) than those in the Au_50 nm_/TiO_2_-HA (ca. 2000 s) and TiO_2_-HA (ca. 300 s). It should be pointed out that the lifetimes of the injected hot electrons in the Au_50 nm_/TiO_2_-HA and electrons in the TiO_2_-HA CB are close to the data reported by DuChene et al..^8^

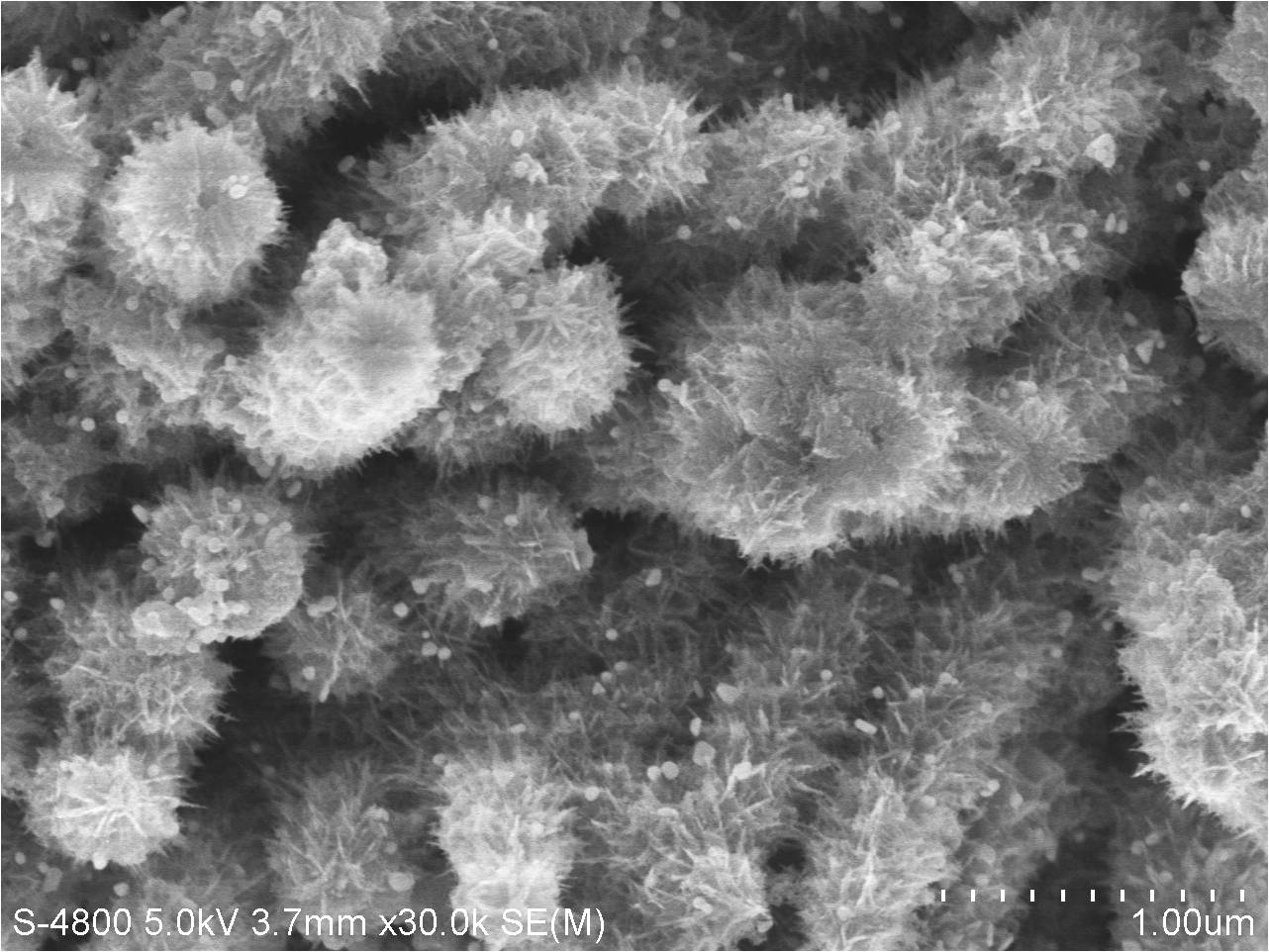

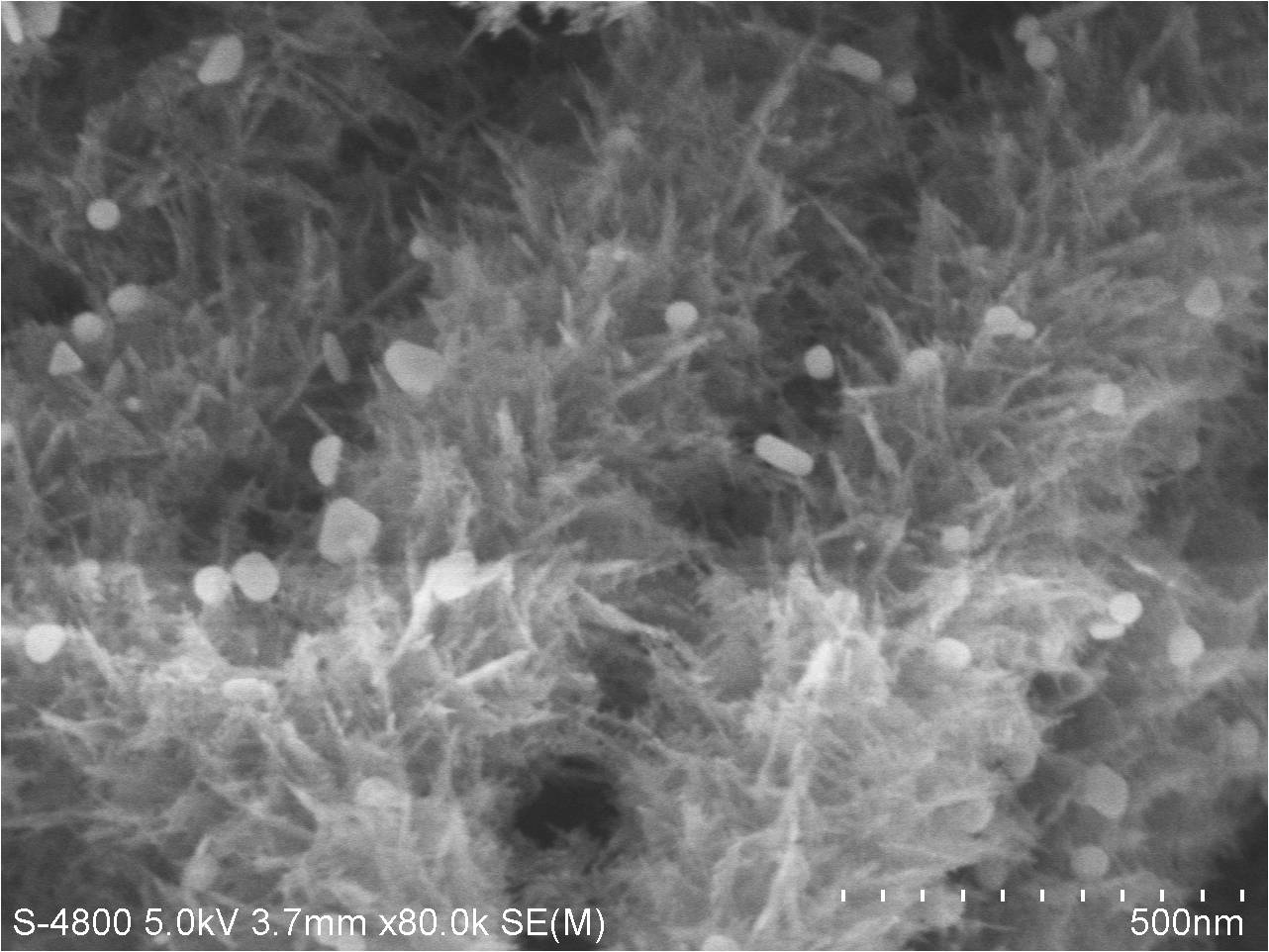

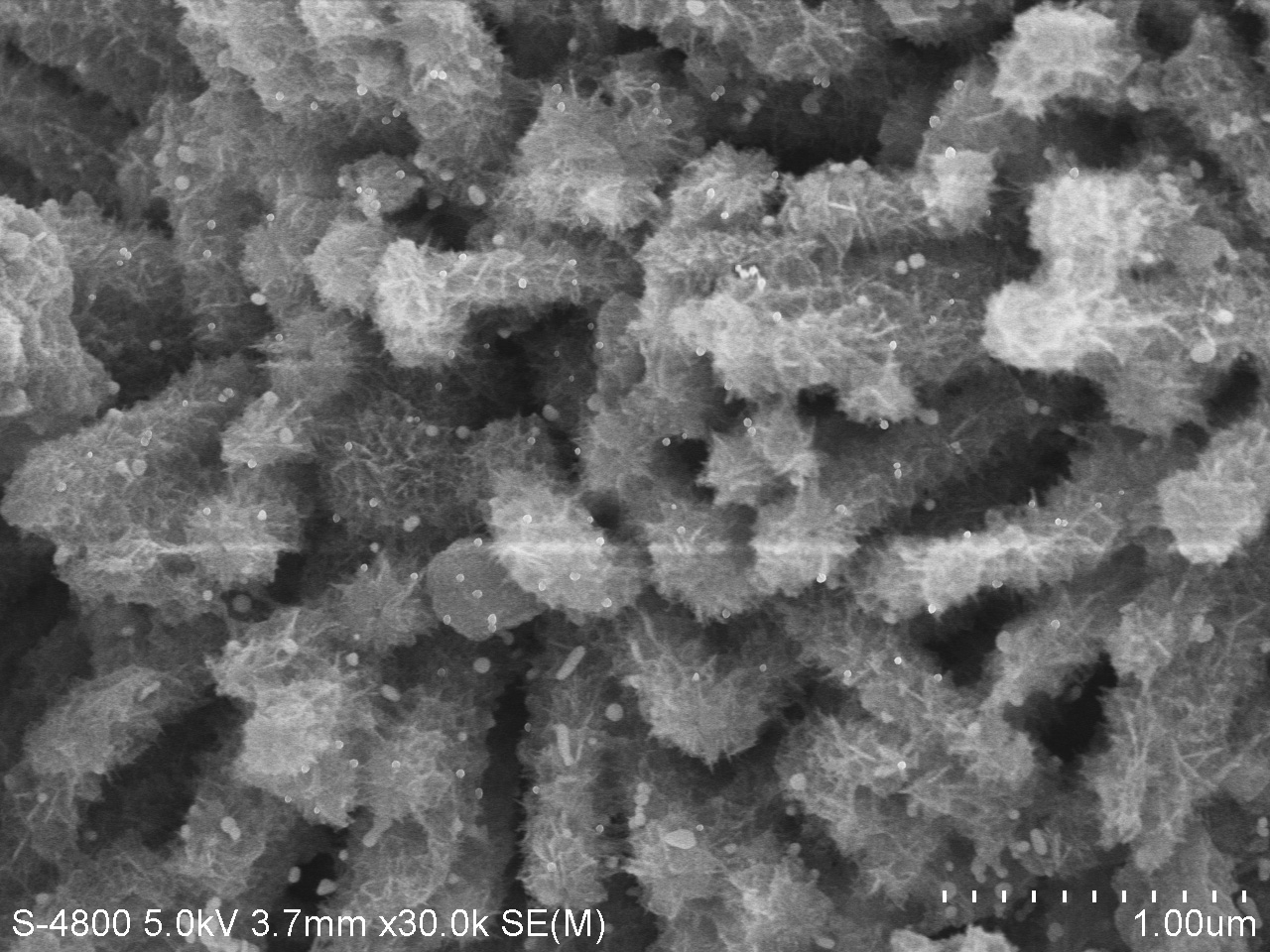

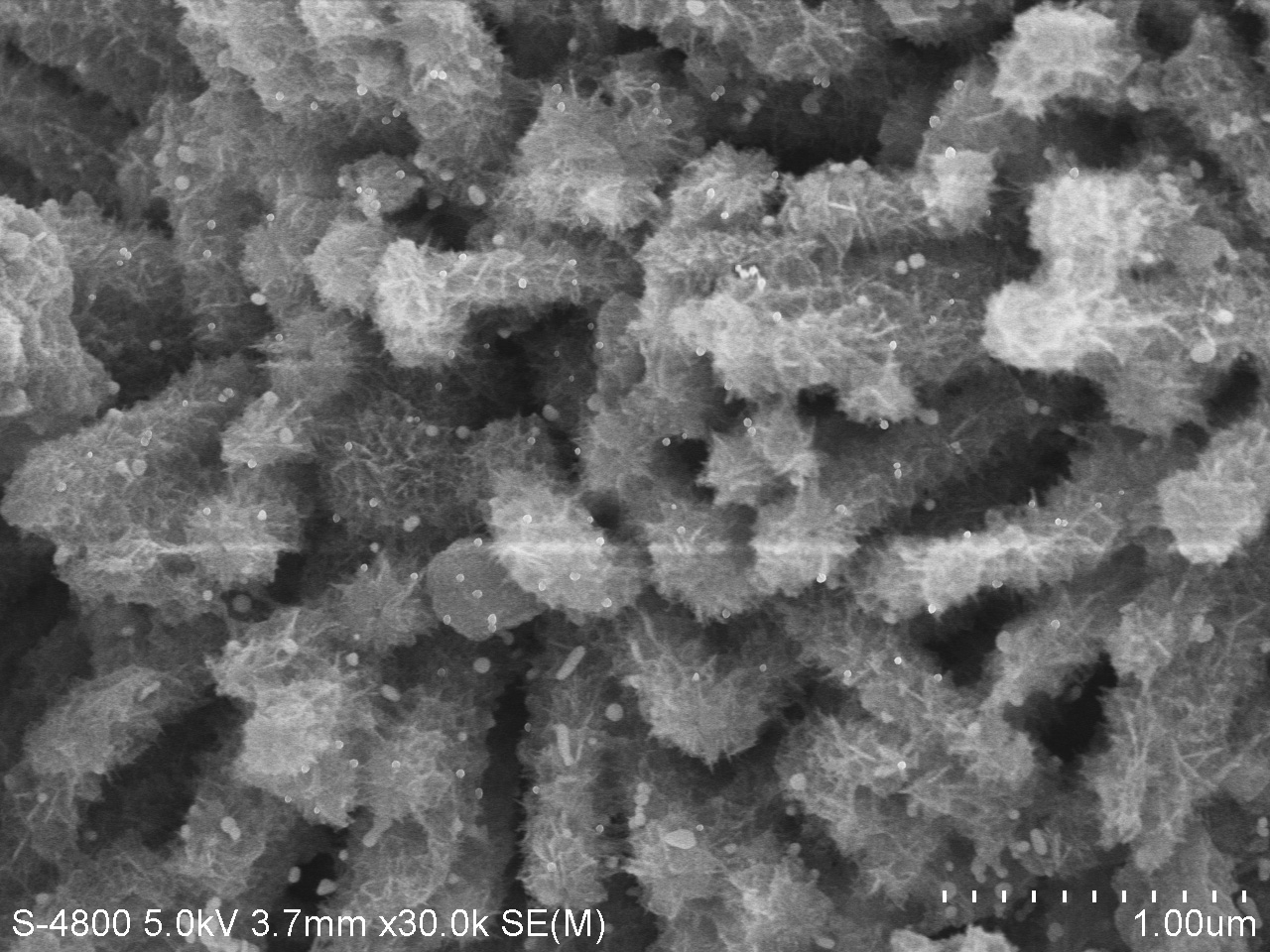


**Figure S9.** (a and c) High- and (b and d) low-magnification SEM images of (a and b) the Au_50 nm_/TiO_2_-HA and (c and d) Au_50 nm_/TiO_2_-HA‒Pt_25 nm_. The orange and cyan arrows in panel (d) indicate Au and Pt NPs, respectively. (e) SEM-EDS spectrum of the Au_50 nm_/TiO_2_-HA‒Pt_25 nm_ sample. The square area marked by a white solid line box in panel (c) is used for recording EDS spectrum. The Si signal arises from the Si substrate for SEM imaging.

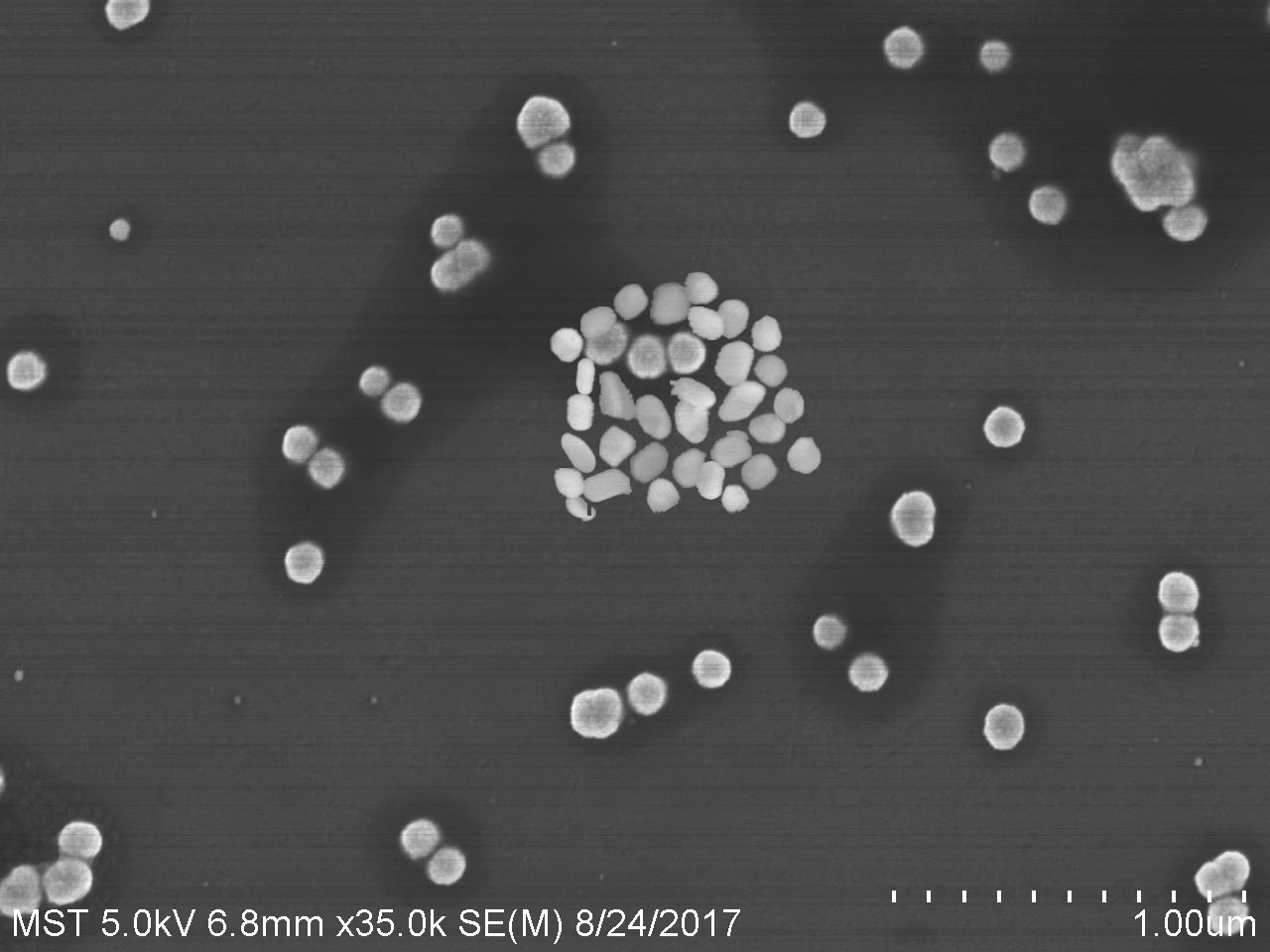


**Figure S10.** (a) XRD pattern and (b) UV-vis extinction spectrum of the Au_50 nm_/TiO_2_-HA and Au_50 nm_/TiO_2_-HA‒Pt_25 nm_. For comparison, the intensities and positions for the pure TiO_2_ (olive bars, JCPDF No. 78-2486), Au (orange bars, JCPDF No. 04-0784), and Pt (cyan bars, JCPDF No. 87-0646) references are given at the bottom in panel (a) according to the JCPDS database. For comparison, the optical extinction spectrum and SEM image of the Au colloids with a similar mean size are also shown in panel (b) and its inset, respectively.

The SEM images of the Au_50 nm_/TiO_2_-HA in Figure S9a,b show that the TiO_2_-HA is decorated with the Au NPs with a mean size of *ca*. 50 nm. The SEM images of the Au_50 nm_/TiO_2_-HA‒Pt_25 nm_ in Figure S9c,d illustrate that the TiO_2_-HA is decorated with both larger Au NPs and small Pt NPs with a mean size of *ca*. 50 and 25 nm, respectively. It can be seen that the noble metal NPs are homogeneously distributed over the TiO_2_-HA. The SEM-EDS spectrum in Figure S9e reveals the Au_50 nm_/TiO_2_-HA‒Pt_25 nm_ comprised of Ti, O, Au, and Pt moieties. For both samples, the weight percentages of Au and Pt NPs determined by the EDS quantitative evaluation are given in Table S1. The XRD patterns of the Au_50 nm_/TiO_2_-HA and Au_50 nm_/TiO_2_-HA‒Pt_25 nm_ in Figure S10a present a set of reflection peaks stemming from the fcc Au NPs and two sets of peaks from fcc Au and Pt NPs, respectively, besides the peaks from the anatase-structured TiO_2_-HA. The above data confirm that Au NPs and both Au and Pt NPs have been incorporated onto the TiO_2_-HA to form the Au_50 nm_/TiO_2_-HA and Au_50 nm_/TiO_2_-HA‒Pt_25 nm_, respectively. Compared to the pristine TiO_2_-HA, a new, strong absorption band centered at 545 nm is observed in the extinction spectra of the Au_50 nm_/TiO_2_-HA and Au_50 nm_/TiO_2_-HA‒Pt_25 nm_ samples (Figure S10b), which is attributed to SPR of the supported Au NPs. Loading the co-catalyst Pt NPs insignificantly changes the extinction spectrum of the Au_50 nm_/TiO_2_-HA‒Pt_25 nm_ while it results in a slight increase in the baseline of the spectrum, invoked by the enhanced light scattering. In contrast, the corresponding Au colloids show the maximum absorption wavelength (*λ*_max_) at 532 nm. Apparently, in the case of the Au_50 nm_/TiO_2_-HA‒Pt_25 nm_ sample, the SPR absorption band red shifts by *ca*. 13 nm after loading of the Au NPs on TiO_2_-HA.

**Figure S11.** (a) Time course of H_2_ volume evolved from an aqueous solution of ethanol (4 : 1 water/ethanol in v/v) and (b) H_2_ and O_2_ evolution from pure water in 3 repeated cycles (8 h/cycle) under visible light (*λ* > 400 nm) irradiation using the Au_50 nm_/TiO_2_-HA and Au_50 nm_/TiO_2_-HA‒Pt_25 nm_ as the photocatalysts. (c) Optical extinction spectrum (left axis, black curve) and action spectrum (left axis, red circle dots) of the Au_50 nm_/TiO_2_-HA‒Pt_25 nm_ photocatalyst in water splitting.

**Figure S12.** The amounts of H_2_ and O_2_ consumption to yield water with time elapse in the dark in the presence of (a) the Pt107-7/TiO_2_-HA and (b) Pt73-7/TiO_2_-HA. The reaction between H_2_ and O_2_ was carried out in a gastight circulation system filled with water (50 mL) and a gas mixture of H_2_ (2%, v/v), O_2_ (1%, v/v), and Ar carrier gas (97%, v/v) under ambient conditions using 10 mg of the Pt107-7/TiO_2_-HA or Pt73-7/TiO_2_-HA as catalyst. The reaction rates of H_2_ are determined to be ~1.4 and ~1.5 μmol h^–1^ for the Pt107-7/TiO_2_-HA and Pt73-7/TiO_2_-HA, respectively.

**Figure S13.** Linear sweep voltammograms of (a) Pt107-7/TiO_2_-HA and (b) Au_50 nm_/TiO_2_-HA‒Pt_25 nm_ film photocathodes recorded in 1 M Na_2_SO_4_ aqueous solution (pH = 5) in the dark and under visible light (*λ* > 420 nm) illumination (~120 mW cm^‒2^). (c) Nyquist plots of the impedance measurements at an applied potential of 0 V vs a reversible hydrogen electrode (RHE) for the Pt107-7/TiO_2_-HA and Au_50 nm_/TiO_2_-HA‒Pt_25 nm_ electrodes under visible light (*λ* > 420 nm) illumination (~120 mW cm^‒2^). The insets in panel (c) show the Nyquist plots of various electrodes on smaller scales (right) and the corresponding equivalent circuit (i.e., a Randles circuit, comprising of a series resistance (*R*_s_), a charge transfer resistance (*R*_ct_), and a constant phase element (CPE)) used to model these two electrode systems (left). (d) Comparison of photocurrent density (*J*‒*V*) curves of Pt107-7/TiO_2_-HA and (b) Au_50 nm_/TiO_2_-HA‒Pt_25 nm_ photoanodes recorded in 1 M Na_2_SO_4_ aqueous solution at pH 10 (borate buffer) to facilitate water oxidation in the dark and under visible light (*λ* > 420 nm) illumination (~120 mW cm^‒2^). The surface morphology and composition of these two film electrodes are nearly the same as those of the corresponding photocatalyst powder. For the sake of clarity and brevity, the characterization dada are not shown again.

**Figure S14.** Long-term stability and durability tests. Photocatalytic H_2_ and O_2_ evolution from pure water over time using an aqueous suspension of (a) the Pt73-7/TiO_2_-HA or (b) Pt107-7/TiO_2_-HA photocatalyst under illumination of visible light obtained from a Xe lamp equipped with an L-42 cut-off filter.

**Table S1.** The designated and measured content of Pt NPs, Au NPs, or both in each photocatalyst.

| Photocatalyst | **The designated content (wt%)** | **The measured Pt content (wt%) by EDS** |
| --- | --- | --- |
| Pt107-7/TiO_2_-HA | 7 | 7.36 |
| Pt107-3/TiO_2_-HA | 3 | 2.93 |
| Pt107-1/TiO_2_-HA | 1 | 1.05 |
| Pt73-7/TiO_2_-HA | 7 | 7.42 |
| Pt73-3/TiO_2_-HA | 3 | 3.34 |
| Pt73-1/TiO_2_-HA | 1 | 1.17 |
| Au_50 nm_/TiO_2_-HA | 7 | 8.34 |
| Au_50 nm_/TiO_2_-HA‒Pt_25 nm_ | 7 for Au and 7 for Pt | 7.84 for Au and 6.79 for Pt |

**Table S2.** Comparison of the average rate (*r*) of H_2_ evolution obtained at 1 h, mass activity, and volume of H_2_ evolved after 6 h ( *V*_6 h_ ) under visible light illumination using different photocatalysts.

| Photocatalyst (mass) | Light source | Medium | *r* (mL h^–1^) | Mass activity*^a^*  (mmol h^–1^ g_C_^–1^) | Mass activity*^b^*  (mmol h^–1^ mg_PM_^–1^) | *V*_4 h_ (mL) |
| --- | --- | --- | --- | --- | --- | --- |
| Pt107-7/TiO_2_-HA (10 mg) | 300 W Xe lamp,  cut-off filter, λ > 400 nm | 4 : 1 v/v water/ethanol | 87.1 | 356 | 4.84 | 197.3 |
| Pt107-3/TiO_2_-HA (10 mg) | 300 W Xe lamp,  cut-off filter, λ > 400 nm | 4 : 1 v/v water/ethanol | 40.3 | 165 | 5.62 | 105.6 |
| Pt107-1/TiO_2_-HA (10 mg) | 300 W Xe lamp,  cut-off filter, λ > 400 nm | 4 : 1 v/v water/ethanol | 12.7 | 51.9 | 4.95 | 36.9 |
| Pt73-7/TiO_2_-HA (10 mg) | 300 W Xe lamp,  cut-off filter, λ > 400 nm | 4 : 1 v/v water/ethanol | 75.0 | 307 | 4.13 | 185.0 |
| Pt73-3/TiO_2_-HA (10 mg) | 300 W Xe lamp,  cut-off filter, λ > 400 nm | 4 : 1 v/v water/ethanol | 33.2 | 136 | 4.06 | 90.5 |
| Pt73-1/TiO_2_-HA (10 mg) | 300 W Xe lamp,  cut-off filter, λ > 400 nm | 4 : 1 v/v water/ethanol | 10.8 | 44.2 | 3.78 | 35.5 |
| Au_50 nm_/TiO_2_-HA (10 mg) | 300 W Xe lamp,  cut-off filter, λ > 400 nm | 4 : 1 v/v water/ethanol | 26.3 | 108 | 1.29 | 68.9 |
| Au_50 nm_/TiO_2_-HA–Pt_25 nm_ (10 mg) | 300 W Xe lamp,  cut-off filter, λ > 400 nm | 4 : 1 v/v water/ethanol | 49.6 | 203 | 2.59 | 124.7 |
| Janus Au 50 nm-TiO_2_ (45 mg) (8.8 wt% Au)^9^ | 500 W tungsten halogen lamp, cut-off filter, λ > 400 nm | 2 : 1 v/v water/isopropyl alcohol | ~65 | ~59 | 0.67 | 138 (*V*_3 h_) |
| Janus Au 70 nm-TiO_2_ (45 mg) (15.0 wt% Au)^9^ | 500 W tungsten halogen lamp, cut-off filter, λ > 400 nm | 2 : 1 v/v water/isopropyl alcohol | ~88 | ~80 | 0.53 | 170 (*V*_3 h_) |
| Janus Au multimer-TiO_2_ (40 mg) (1.8 wt% Au)^10^ | 300 W Xe arc lamp,  cut-off filter, λ > 420 nm | 7 : 3 v/v water/methanol | ~25 | ~26 | 1.4 | ~118 (*V*_8 h_) |

*^a^* The mass activity is calculated based on the total mass of each photocatalyst. *^b^* The mass activity is calculated based on the actual mass of the plasmonic metal supported on each photocatalyst, determined by EDS quantitative analysis.

**Table S3.** Comparison of the rates of H_2_ evolution (*r*_H2_) and O_2_ evolution (*r*_O2_) over a different photocatalyst in pure water and the corresponding mass activity under visible-light irradiation obtained from a Xe lamp (300 W).

| Photocatalyst  (10 mg in this work) | Irradiation wavelength | *r*_H2_ (μmol h^–1^) | *r*_O2_ (μmol h^–1^) | Mass activity*^b^*  (μmol h^–1^ g^–1^) | Mass activity*^c^*  (μmol h^–1^ mg_PM_^–1^) |
| --- | --- | --- | --- | --- | --- |
| Pt107-7/TiO_2_-HA | *λ* > 400 nm | 25.2*^a^* | 12.9*^a^* | 2521 | 34.3 |
|  | *λ* > 420 nm | 24.6*^a^* | 12.5*^a^* | 2461 | 33.4 |
| Pt107-3/TiO_2_-HA | *λ* > 400 nm | 11.6*^a^* | 5.8*^a^* | 1159 | 39.6 |
| Pt107-1/TiO_2_-HA | *λ* > 400 nm | 3.5*^a^* | 1.8*^a^* | 349 | 33.2 |
| Pt73-7/TiO_2_-HA | *λ* > 400 nm | 17.6*^a^* | 8.8*^a^* | 1755 | 23.7 |
|  | *λ* > 420 nm | 16.4*^a^* | 8.1*^a^* | 1638 | 22.1 |
| Pt73-3/TiO_2_-HA | *λ* > 400 nm | 7.6*^a^* | 3.8*^a^* | 756 | 22.6 |
| Pt73-1/TiO_2_-HA | *λ* > 400 nm | 2.3*^a^* | 1.2*^a^* | 234 | 20.0 |
| Au_50 nm_/TiO_2_-HA | *λ* > 400 nm | 0 | 0 | 0 | 0 |
| Au_50 nm_/TiO_2_-HA–Pt_25 nm_ | *λ* > 400 nm | 0.54*^a^* | 0.27*^a^* | 54 | 0.65 |
| Au(1.0 wt%)/TiO_2_-NiO_x_(0.5 wt%)^11^ | *λ* > 420 nm | 5.5 | 2.8 | 18 | 1.8 |
| Au nanorods/TiO_2_/  Pt/Co-OEC^12^ | *λ* > 410 nm | 0.25 ± 0.05 | no data | 1140 | no data |

*^a^* The values are obtained by averaging the data from three cycles (8 h/cycle). *^b^* The mass activity is calculated based on the moles of H_2_ production and the total mass of each photocatalyst. *^c^* The mass activity is calculated based on the actual mass of the plasmonic metal supported on each photocatalyst, determined by EDS quantitative analysis.

**Table S4.** Best-fit estimates of the equivalent circuit parameters obtained from the impedance measurements

| parameter | Pt107-7/TiO_2_-HA | Au_50 nm_/TiO_2_-HA‒Pt_25 nm_ |
| --- | --- | --- |
| *χ*^2^ | 4.85 × 10^–4^ | 3.08 × 10^–3^ |
| *R*_s_ (Ω cm^‒2^) | 15.0 ± 0.1 | 86.2 ± 1.4 |
| *R*_ct_ (Ω cm^‒2^) | (16.7 ± 0.1) × 10^3^ | (30.6 ± 1.1) × 10^3^ |
| *CPE*(*T*) | (4.11 ± 0.03) × 10^–3^ | (2.51 ± 0.08) × 10^–3^ |
| *CPE*(*P*) | 0.712 ± 0.001 | 0.776 ± 0.004 |
| *C*_dl_ (mF cm^‒2^) |  |  |

**References**

1. Berkó, A.; Ulrych, I.; Prince, K. C. *J. Phys. Chem. B* **1998**, *102*, 3379–3386.
2. Zhang, J.; Yan, S.; Fu, L.; Wang, F.; Yuan, M.; Luo, G.; Xu, Q.; Wang, X.; Li, C. *Chin. J. Catal*. **2011**, *32*, 983–991.
3. Pfeiffer, H.; Tancret, F.; Brousse, T. *Electrochim. Acta* **2005**, *50*, 4763–4770.
4. Dai, Z.; Geng, H.; Wang, J.; Luo, Y.; Li, B.; Zong, Y.; Yang, J.; Guo, Y.; Zheng, Y.; Wang, X.; Yan, Q. *ACS Nano* **2017**, *11*, 11031–11040.
5. Yu, J. C.; Zhang, L.; Zheng, Z.; Zhao, J. *Chem. Mater*. **2003**, *15*, 2280–2286.
6. Ni, J.; Fu, S.; Yuan, Y.; Ma, L.; Jiang, Y.; Li, L.; Lu, J. *Adv. Mater*. **2018**, *30*, 1704337.
7. Chen, J.; Liu, Z.; Wu, Y.; Li, Y.; Zhao, J.; Zhu, X.; Na, P. *Chem. Commun*. **2018**, *54*, 1972–1975.
8. DuChene, J. S.; Sweeny, B. C.; Johnston-Peck, A. C.; Su, D.; Stach, E. A.; Wei, W. D. *Angew. Chem. Int. Ed*. **2014**, *53*, 7887–7891.
9. Seh, Z. W.; Liu, S.; Low, M.; Zhang, S.-Y.; Liu, Z.; Mlayah, A.; Han, M.-Y. *Adv. Mater*. **2012**, *24*, 2310–2314.
10. Zhang, J.; Jin, X.; Morales-Guzman, P. I.; Yu, X.; Liu, H.; Zhang, H.; Razzari, L.; Claverie, J. P. *ACS Nano* **2016**, *10*, 4496–4503.
11. Tanaka, A.; Teramura, K.; Hosokawa, S.; Kominami, H.; Tanaka, T. *Chem. Sci*. **2017**, *8*, 2574–2580.
12. Mubeen, S.; Lee, J.; Singh, N.; Kraemer, S.; Stucky, G. D.; Moskovits, M. *Nat. Nanotechnol*. **2013**, *8*, 247–251.
